# Automated Sound Recognition Provides Insights into the Behavioral Ecology of a Tropical Bird

**DOI:** 10.1371/journal.pone.0169041

**Published:** 2017-01-13

**Authors:** Olaf Jahn, Todor D. Ganchev, Marinez I. Marques, Karl-L. Schuchmann

**Affiliations:** 1 National Institute for Science and Technology in Wetlands (INAU), Science without Borders Program, Federal University of Mato Grosso (UFMT), Cuiabá, Mato Grosso, Brazil; 2 Zoological Research Museum A. Koenig (ZFMK), Bonn, North Rhine-Westphalia, Germany; 3 Department of Computer Science and Engineering, Technical University of Varna, Varna, Varna, Bulgaria; 4 Institute of Biosciences, UFMT, Cuiabá, Mato Grosso, Brazil; 5 University of Bonn, Bonn, North Rhine-Westphalia, Germany; Virginia Commonwealth University, UNITED STATES

## Abstract

Computer-assisted species recognition facilitates the analysis of relevant biological information in continuous audio recordings. In the present study, we assess the suitability of this approach for determining distinct life-cycle phases of the Southern Lapwing *Vanellus chilensis lampronotus* based on adult vocal activity. For this purpose we use passive 14-min and 30-min soundscape recordings (n = 33 201) collected in 24/7 mode between November 2012 and October 2013 in Brazil’s Pantanal wetlands. Time-stamped detections of *V*. *chilensis* call events (n = 62 292) were obtained with a species-specific sound recognizer. We demonstrate that the breeding season fell in a three-month period from mid-May to early August 2013, between the end of the flood cycle and the height of the dry season. Several phases of the lapwing’s life history were identified with presumed error margins of a few days: pre-breeding, territory establishment and egg-laying, incubation, hatching, parental defense of chicks, and post-breeding. Diurnal time budgets confirm high acoustic activity levels during midday hours in June and July, indicative of adults defending young. By August, activity patterns had reverted to nonbreeding mode, with peaks around dawn and dusk and low call frequency during midday heat. We assess the current technological limitations of the *V*. *chilensis* recognizer through a comprehensive performance assessment and scrutinize the usefulness of automated acoustic recognizers in studies on the distribution pattern, ecology, life history, and conservation status of sound-producing animal species.

## Introduction

Habitat conversion, overexploitation, climate change, and other human-induced environmental factors increasingly affect global populations of vertebrates [1]. Freshwater species have been suffering the biggest losses, with 81% of populations declining from 1970 to 2012 [2]. Nevertheless, considering the global situation of inland waters, the world still seems to be in good condition in our study area, Brazil’s Pantanal wetlands. However external factors, like climate change, the construction of hydroelectric dams, gold mining, erosion, and the use of agrochemicals in the adjacent *Cerrado* region are threatening the ecosystem [3,4].

To understand the ongoing environmental changes in the Pantanal careful monitoring of multiple factors is required [5]. In an effort to establish a remote monitoring system for sound-producing animal species, we used autonomous recording units to collect soundscapes in the northern Pantanal region [6]. In comparison with classical survey methods [7,8], automated acoustic surveillance has many advantages [9]. For example, automated acoustic monitoring (a) can be used for studying various taxonomic groups of sound-producing animals continuously and independently of the time of day [10–14]; (b) allows the detection of elusive, rare, and threatened species [15,16]; (c) facilitates temporary inventories and long-term monitoring studies [17–19]; (d) supports presence/absence surveys as well as the estimation of species richness [20], population density [19,21–24], and bird colony size [25]; (e) is suitable for virtually all terrestrial and aquatic habitats, including vast and remote areas and rough terrain [26–28]; (f) causes minimal disturbance to the surveyed animal communities and ecosystems [29]; and (g) provides a permanent record of detections that can be archived, disseminated, and re-assessed by peers [30]. Furthermore, sound recordings can be wirelessly transmitted via existing mobile communication infrastructure or custom-built networks [31] and analyzed remotely [18,32–36].

Passive audio recordings have been used to determine vocal activity patterns of animal species for many years. Earlier studies had in common that activity was analyzed by hearing the recordings and annotating the detections manually [37–41]. More recently, the emergence of computational bioacoustics has allowed the automated screening of soundscapes with sound recognizers and has facilitated studies on acoustic activity patterns of selected species, including insects, anurans, birds, and mammals [17–19,42,43].

Here we examine the suitability of automated species recognition for the characterization of certain life-cycle phases of the Southern Lapwing *Vanellus chilensis*, a widespread and regionally common Neotropical shorebird [44,45]. We selected the ground-dwelling lapwing as a study object because it is vocally active all months of the year and rather well studied. Specifically we assess the hypothesis that distinct phases of the *V*. *chilensis* life cycle, such as territory establishment, egg-laying and incubation, hatching, adults tending young, and post-breeding, can be determined by differences in the intensity of adult lapwing acoustic activity. In order to detect the time-varying sound emissions of the lapwing we used an automated species-specific recognizer for *V*. *chilensis* [36] to process 12 months of continuous soundscapes from a single recording station. Since our work is based on passive recordings, i.e. without accompanying field observations, we needed a clear theoretical framework for a meaningful analysis of *V*. *chilensis* vocal activity patterns. The backbone of our interpretations is the work of Walters & Walters [46] and Walters [47–49], who studied 20 *V*. *chilensis* reproductive social units (RSU; *cf*. [50]) during the main breeding season in the Venezuelan Llanos. Important additional data on the social structure and behavior of breeding lapwings were gathered during biannual studies in Central Brazil’s *Planalto* region by Saccura et al. [51] (n = 36 RSUs) and Santos & Macedo [50] (n = 74 RSUs). These studies show that *V*. *chilensis* breeds either in monogamous pairs or cooperatively in groups of 3–4 birds [46,50,51]. Both sexes engage in territory defense, incubation, and caring for their precocial and nidifugous chicks [47]. The species is extremely vigilant with respect to both intraspecific intruders and potential predators. Most importantly for our study, vocalizations are part of the majority of behavioral responses and pronounced differences in behavioral patterns exist between territorial nonbreeding and breeding *V*. *chilensis* groups, with a distinct increase in parental defense behavior from early to late incubation [46–49,52]. The frequency of attacks on predators and interspecific intruders reaches maximum levels around chick hatching and gradually decreases until young lapwings acquire the ability to fly and get independent. This pattern is in agreement with the prediction that in precocial birds, chick vulnerability peaks at hatching due to the subsequent increase in chick mobility [53]. Therefore we make four assumptions about the seasonality of adult *V*. *chilensis* acoustic activity patterns at our Pantanal recording site (*cf*. section Assumptions on the seasonality of vocal activity). *A1*: Vocal activity of nonbreeding territorial lapwings fluctuates around an average value.—Southern Lapwing RSUs and territories are rather stable [50,51] so that temporal changes in vocal activity are mainly due to intrusions of nonbreeding floaters [46] and response of territory holders to certain predators [49]. *A2*: Vocal activity decreases during periods of flooding.—Extensive flooding causes lapwings and other charadriids to perform local movements (*cf*. [46],[54]: p. 222) resulting in low calling activity levels in inundated areas. *A3*: Adult vocal activity increases due to parental defense and interspecific aggression during incubation, reaches its highest levels at or shortly after hatching, and gradually decreases thereafter [46–49]. *A4*: An all-day-active mode is energetically costly and risky for adults and thus characteristic for lapwings tending young.—In order to defend their chicks from predators and interspecific competitors, adults are vocally active all day (*cf*. [47],[48]: p. 253). By contrast, nonbreeding birds are mostly inactive during midday hours [55], supporting the presumption that the state of permanent activity is costly for adults.

Here we demonstrate how assumptions *A1-A4* can be used to determine distinct *V*. *chilensis* life cycle phases based on automatically detected adult acoustic activity. We carried out a recognizer performance assessment to validate our interpretations. Despite several identified biases, the recognizer output supports our hypothesis.

## Materials and Methods

### Ethics statement

This project is run under the coordination of *KLS* and *MIM* (Instituto Nacional de Ciência e Tecnologia em Áreas Úmidas (INAU); ‘Programa Ciência sem Fronteiras’ (CsF) of the Brazilian government). Research was conducted under permission of the Brazilian Ministério do Meio Ambiente (MMA), Instituto Chico Mendes de Conservação da Biodiversidade (ICMBio), Sistema de Autorização e Informação em Biodiversidade (SISBIO, permit no 39095–4 issued to *KLS*). Automated acoustic monitoring is a non-invasive survey method that does not negatively affect the studied animal communities.

### Study area and data acquisition

Our study was carried out in the northern Pantanal region, municipality of Poconé, Mato Grosso, Brazil. Audio recordings were collected with Song Meter SM2+ recorders (Wildlife Acoustics; www.wildlifeacoustics.com) in the Fazenda Pouso Alegre area (–16.50303 S, –56.74533 W; 115–126 m a.s.l.; *c*. 110 km^2^; recording period: July 2012 through October 2013) and at the Estância Ecológica SESC Pantanal: Reserva Particular do Patrimônio Natural, Baía das Pedras (–16.49879 S, –56.41309 W; 119–131 m a.s.l.; 878.7 km^2^; recording period: November 2013 until June 2016). In total we gathered *c*. 120 TB of continuous soundscapes.

For the purpose of the present research, we automatically analyzed the acoustic activity of *Vanellus chilensis* for the period 29 October 2012 to 26 October 2013, i.e. 12 months of recordings. As of 6 October 2013 we gathered 32 242 soundscapes of 14 min, with intervening pauses of 1 min. Moreover, until the end of the recording period at Fazenda Pouso Alegre we gathered another 959 soundscapes of 30 min without pauses. Very few gaps in time coverage occurred due to logistical problems: *c*. 4 days around New Year’s Eve 2012/2013, 27 h between 11 and 12 January 2013, and 20.5 h between 17 and 18 August 2013. Eventually, we performed a comprehensive data analysis only for April to September 2013 (section Validation datasets and assessment procedures). The latter subsample contained 17 493 soundscapes of 14 min each (*c*. 2.7 TB = 4373 h).

All data originate from PPA001, one of nine permanent recording stations deployed at the property of Fazenda Pouso Alegre. PPA001 was located in an extensive, seasonally-flooded, and savannah-like cattle pasture (S1 and S2 Figs). In 2013 the flooding persisted between mid-January and mid-June, whereas in other years floodwaters may recede as early as March. Starting with 1 April, we took water level measurements on every seventh day until 9 June, i.e. on 11 occasions until the floodwaters completely receded. We measured the water level at a distance of 300 m from PPA001, after readings taken at both sites demonstrated that they were almost identical.

The Song Meter SM2+ recorder was configured for 48 dB hardware amplification and 6 dB software amplification and we used two-channel recording with sampling rate 48 kHz, resolution 16 bits per sample, and no WAC compression. Here we processed only channel A, corresponding to the left microphone (i.e. omnidirectional SMX-II). We did not explicitly measure the perceptive range of the SM2+ recorder. Likewise, information about the source level and directionality is not available. Since the noise floor level varied over time due to changing environmental conditions (e.g., S1 and S2 Figs), the detection distance for *V*. *chilensis* sound events also varied. However, the signal amplitude of the weakest *V*. *chilensis* calls detected in the soundscapes showed little variation in the study period (section Recognizer performance assessment). Therefore we regard the effect of the time-varying ambient noise as negligible. We estimate that the weakest *V*. *chilensis* signals recorded in this study (*c*. –50 dB), correspond to a distance of 300 and 500 m from the microphone. This was the minimum distance between station PPA001 and some higher-ground areas, such as dirt roads and cattle gathering sites, which had already fallen dry in early April (*cf*. S1A Fig, showing the extent of drying open areas in pinkish color as of 4 May).

### The target species

Southern Lapwing *Vanellus chilensis* (Molina, 1782) is a common and widespread Neotropical wader of open habitats, such as savannahs, steppes, floodplains, riverbanks, lakeshores, boggy terrain with short matted vegetation, pastures, city parks, and ploughed fields. Its four recognized subspecies are distributed from Nicaragua south to Tierra del Fuego, Argentina [44,45,56,57]. The subspecies present in Central Brazil is *V*. *c*. *lampronotus* (Wagler, 1827) (S3 Fig).

#### Vocalizations

**Fig 1:**
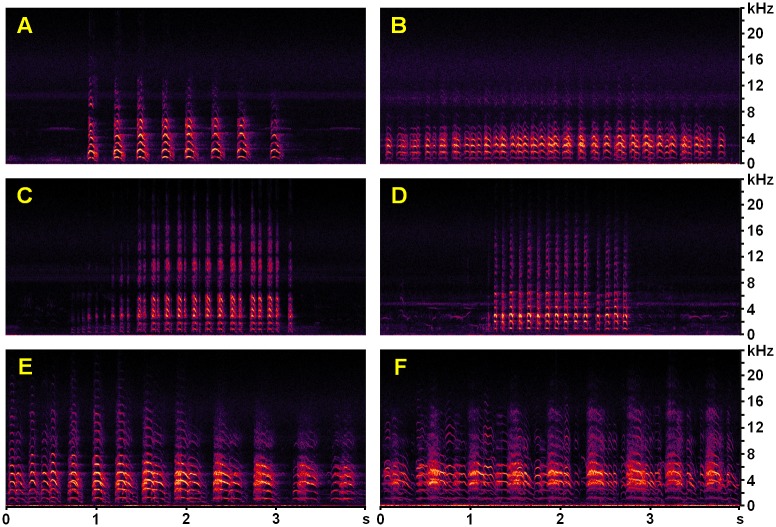
Spectrograms of exemplary *Vanellus chilensis* vocalizations. All spectrograms represent unfiltered 4-s segments of WAV soundscapes passively recorded at station PPA001 in the Fazenda Pouso Alegre area (windowing function: Blackman-Harris; resolution: 512 bands; decibel range: 85 dB; the strongest target signals were equalized to –3 dB). A: flight calls of a lapwing patrolling its inundated territory; 28 Apr. 2013, 17:38 h (S1 Audio). B: display and agitated cackling of a pair flying over their inundated territory; 29 Apr. 2013, 16:35 h (S2 Audio). C: short series of cackling alarm calls of perched *V*. *chilensis* close to its nest; 31 May 2013, 14:31 h (S4 Audio). D: short series of agitated alarm calls of a lapwing flushed from its nest or close to it; 30 May 2013, 06:13 h (S6 Audio). E: excerpt of a long alarm call series of a *V*. *chilensis* flock mobbing a predator to defend their chicks; 16 Jun. 2013, 15:35 h (S8 Audio). F: segment of a long alarm call series of a pair or flock mobbing a potential predator or food competitor; 21 Jun. 2013, 12:28 h (S9 Audio).

Southern Lapwing is vocally active all months of the year and at day and night [44,54,58]. Their sounds are particularly conspicuous during flapping courtship and territorial flight displays as well as in threat situations [36,49]. At least some of the variation in alarm calls is functional [48,49]. Choruses are frequently heard during the nonbreeding season, when flocks up to a few hundred birds congregate [44,46,55,59]. The spectral structure of typical call notes emitted by adult and immature birds is quite complex and consists of multiple harmonics with most of the energy concentrated in the frequency range 1.1–10 kHz (*cf*. [36]: Fig 1, Table 1). We point out that only the aforementioned vocalization types were available for the compilation of the *V*. *chilensis* training library ([36]: section 2.1.3.2) used for detector development (section The automated acoustic detector). Other less well-known lapwing call variants undoubtedly exist, including distinct short series of dry and mechanical-sounding warning rattles, low-volume/low-frequency contact calls lacking the characteristic upper harmonics of other vocalizations of adults, and calls emitted by chicks. Because the latter call types were not represented in the *V*. *chilensis* training library, they were neither detected by the automated sound recognizer nor considered in the analyses of the manually annotated validation library (section Validation datasets and assessment procedures). Explicitly, in the present study we focus only on the characteristic multi-harmonic lapwing vocalizations (Fig 1, S1–S9 Audios; and [36]: Fig 1).

**Table 1:** Cumulated monthly number and precision of *Vanellus chilensis* raw detections.

Year	Month	Raw detections	Precision (%)^1^
**2012**	November	4429	
**2012**	December	2650	
**2013**	January	3184	
**2013**	February	3556	
**2013**	March	2825	
**2013**	April	3159	48.0
**2013**	May	7663	72.6
**2013**	June	18 151	91.9
**2013**	July	8899	97.0
**2013**	August	4078	92.6
**2013**	September	1642	68.7
**2013**	October	2056	
**2012/2013**	Total all months	62 292	
**2013**	Total Apr. to Sep.	48 879	

^1^The precision was determined for the period April to September, covering the supposed *V*. *chilensis* main breeding season. See S1 Table for details on the calculation of precision.

#### Assumptions on the seasonality of vocal activity

Here we present a detailed account on the bibliographic support for the assumptions used in the interpretation of the automatically detected *V*. *chilensis* sound events (*cf*. Introduction), which in the text are referred to as *A1-A4*.

*A1*: Vocal activity of nonbreeding territorial lapwings fluctuates around an average value.—The composition and size of reproductive social units (RSUs) show little variation and the birds usually return to the same breeding territory each year [50,51]. While stable group size and territory siting should mean less intraspecific conflicts, intrusions of strangers are frequent in areas with high population densities [46]. Territory holders fiercely attack nonbreeding floaters [46], causing temporary increases in calling frequency. Likewise, *V*. *chilensis* responsiveness to natural predators is generally low outside the breeding season, except against caracaras and humans, which always provoke noisy alarm response [49]. Consequently, we assume short-term variations in otherwise steady acoustic activity levels are typical for nonbreeding territorial lapwings.

*A2*: Vocal activity decreases during periods of flooding.—Flooding weakens territory defense [46]. However, some RSUs may briefly visit their territories even when the area is completely inundated [46]. Eventually, extensive flooding causes lapwings and other charadriids to abandon their territories and to perform local movements (*cf*. [54]: p. 222). Therefore, in inundated areas *V*. *chilensis* calling frequency should reach its lowest level at the height of the flood cycle and increase again as soon as waters recede and territories are reestablished.

*A3*: Adult vocal activity increases due to parental defense and interspecific aggression during incubation, reaches its highest levels at or shortly after hatching, and gradually decreases thereafter.—Southern Lapwings caring for young have a large repertoire of danger-response behaviors. Most responses include warning and alarm calls, specifically (a) alerting call and vigilant posturing for distant threats; (b) reptile-specific alarm call and pecking attack; (c) alarm call, swooping attack, injury-feigning, and mobbing against non-reptilian predators; and (d) “ungulate display” of incubating birds towards cattle ([49]: p. 54; [60]). Less vocal behaviors, like crouched run and false brooding may be an integral part of the latter three defense strategies. While responsiveness to natural predators is generally low in nonbreeding *V*. *chilensis* [49], the frequency of parental defense increases in incubating birds, and is most intense in adults defending small chicks [46–49,52].

Territorial defense behavior also intensifies in the breeding period. Besides lapwings from neighboring territories mob predators together [61], territory holders will chase conspecifics away as soon as the threat ceases [49]. Moreover, another form of parental care, occurring most frequently in adult lapwings with downy chicks, is aggression towards other species of non-predatory birds entering their territories [47]. Adults may launch 10 to 20 interspecific attacks per hour and RSU. Parental care and defense behavior continues for at least six to eight weeks until the chicks fledge [46–48], meaning that adult acoustic activity decreases only slowly after hatching. Taking into account that most *V*. *chilensis* behavioral responses are accompanied by vocalizations [47–49], we predict a roughly bell-shaped pattern of adult acoustic activity during the breeding season.

*A4*: An all-day-active mode is energetically costly and risky for adults and thus characteristic for lapwings tending young.—Compared with the nonbreeding stage (*cf*. [55]), breeding Southern Lapwings spend less time loafing, preening, or sitting [47,48]. After hatching the effort invested in parental care increases further so that adults spend considerably less time foraging than nonbreeding or incubating birds ([47]: p. 1033). One consequence of the increasing reproductive effort is that adults with chicks are vocally active all day (*cf*. [48]: p. 253). By contrast, nonbreeding lapwings are mostly inactive during midday hours [55], supporting the hypothesis that the all-day active mode is costly for parents.

#### Duration of the breeding cycle

In order to put the expected increase in vocal activity in breeding lapwings into a temporal context it is crucial to know the typical duration of each phase of the breeding cycle as precisely as possible (*cf*. assumptions *A3* and *A4*). According to the literature, a complete and successful reproductive cycle of *V*. *chilensis* takes about 3.5 to 5 months from egg-laying to offspring independence. Clutch size in Southern Lapwing is 2–4 eggs, 3 eggs on average (*cf*. [62]: p. 307; [46]: 2.9 ± 0.5, n = 15 clutches; [50]: 3.2 ± 0.7, n = 74; and [63]: n > 100), with larger clutches being an exception [46,63]. Despite laying intervals not being reported in the literature, they can be inferred to be 1–3 days from available data on other vanelline plovers, such as Northern Lapwing *V*. *vanellus* (*cf*. [56]: 30–48 h and [64]: 2.8 days) and Red-wattled Lapwing *V*. *indicus* ([65]: 1 day). Net incubation time is 26–27 days [52,66], corresponding to about 30 days including egg-laying [46]. Chicks retain downy plumage for 3–4 weeks and fledging occurs at 6–8 weeks ([47]: n = 5 broods with 1–3 chicks; [49]). Juveniles join their parents for another 4–8 weeks after fledging [47]. Thus combining this benchmark data on the duration of each phase of the reproductive cycle with the information on the intensity of parental and territorial defense in *V*. *chilensis* [46–49,52], we anticipate the following pattern of acoustic activity during a typical breeding cycle. Calling frequency increases soon after egg-laying for a period of about 3–4 weeks and peaks at or shortly after hatching. Intense vocal activity continues for several days or a few weeks before gradually decreasing during a prolonged period of up to two months until chicks become independent.

### The automated acoustic detector

**Fig 2:**
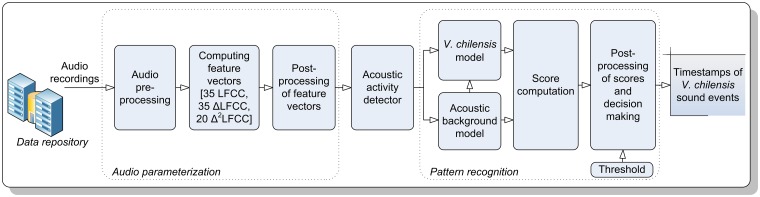
Overall block diagram of the *Vanellus chilensis* recognizer, a Gaussian Mixture Model—Universal Background Model (GMM-UBM)-based detector.

For the automated recognition of *Vanellus chilensis* vocalizations we used the species-specific acoustic detector reported in Ganchev et al. [36]. The automated species-specific detector consisted of two main processing stages, audio parameterization and pattern recognition (Fig 2).

The audio parameterization consisted of audio preprocessing, feature extraction, and feature standardization steps. The preprocessing comprised: (i) rescaling the amplitude of each soundscape recording to peak levels [–1, +1] through adaptive gain control (AGC) amplified weak audio events that otherwise would be disregarded due to their low energy; (ii) down-sampling the signal to sampling rate 24 kHz reduced the computational demands in the subsequent processing steps and removed high-frequency insect sounds ≥ 12 kHz; (iii) filtering with a Butterworth high-pass filter (order 10, cut-off frequency 1000 Hz) suppressed low-frequency interference, such as wind noise and some anthropogenic noise of distant cars and airplanes; all remaining sound signals were treated uniformly regardless of their origin; and (iv) segmenting the audio to short 20-ms segments with 75% overlap.

For each segment we computed a set of 90 audio descriptors, including 35 Linear-Frequency Cepstral Coefficients (LFCC) [67] and their first and second temporal derivatives, referred to as ΔLFCC and Δ^2^LFCC respectively. Here we kept only the first 20 Δ^2^LFCCs, as the last 15 were found to be less informative. All feature vectors were post-processed to uniform dynamic range in order to facilitate the pattern recognition process. During the signal parameterization, we treated all target sounds and noises uniformly, so that the abovementioned set of 90 audio descriptors was computed for each segment of audio. Based on the post-processed feature vectors, an acoustic activity detector selects for further processing only those parts of the audio with considerable acoustic activity. Here we set the threshold to –50 dB with respect to the full range input of the SM2+ recorder. The selected segments are used in the pattern recognition process. We only considered detections of acoustic events with amplitudes of at least –50 dB because below that level *V*. *chilensis* signals were to faint for being discerned and annotated with confidence, even for a bird sound expert (section Recognizer Performance Assessment).

The *V*. *chilensis* detector used in this study explicitly modelled the target species and the acoustic environment through purposely developed Gaussian Mixture Models (GMMs). In particular, based on 75 h of soundscape recordings, we built a Universal Background Model (UBM) via an Expectation–Maximization (EM)-based training of a 512-component GMM. The UBM represents the model of everything that is not Southern Lapwing. Next, we created the *V*. *chilensis* model via means-only adaptation of the UBM and a purposely-designed training dataset, containing characteristic lapwing vocalization types (section The target species). The training dataset consisted of manually tagged and selectively filtered recordings of the target species ([36]: section 2.1.3.2). Subsequently, each vector of audio descriptors is scored against both models and the degree of similarity is computed. The log-likelihood estimator scores were compared with a predefined threshold in order to make decision on the presence or absence of *V*. *chilensis* vocalizations in the particular audio segment. Based on this decision, we assigned each audio segment represented by a set of audio descriptors to the target species or to the background noise. In this sense, we treated audio segments containing sounds of wind, rain, and other interferences no differently than any other portion of audio. The only difference was that they showed lower scores when tested against the target species model and, on some occasions, higher scores when tested against the UBM. Therefore segments dominated by wind, rain, and other non-target noises were typically classified as not representing *V*. *chilensis* acoustic activity.

In a last step, we processed the sequence of scores and decisions with a temporal smoothing algorithm that incorporates domain knowledge about the structure and duration of the target *V*. *chilensis* sound events. Similar to Ganchev et al. ([36]: Table 2), we implemented a set of simple rules: (a) the minimum duration of a call was 50 ms and (b) the maximum duration of pauses between calls in a call series was arbitrarily set at 0.6 s. The first rule translated to convolution of the sequence of decisions with a 50-ms window function that smoothed fragmentation due to interferences in the detection of individual calls, facilitating reliable reconstruction of the start and end of *V*. *chilensis* call events. The second rule led to convolution of single calls with a window function of duration 600 ms, which ideally resulted in detection of cohesive sequences of calls as a single call series.

**Table 2:** Validation library: cumulated number of confirmed and unconfirmed *Vanellus chilensis* call events according to the loudest call amplitude in the vocalizations.

Range (dB)	N target^1^	N unconfirmed	N total	% unconfirmed
0 to –5	0	0	0	NA
0 to –10	6	0	6	0
0 to –15	10	0	10	0
0 to –20	31	0	31	0
0 to –25	55	0	55	0
0 to –30	103	0	103	0
0 to –35	179	1	180	0.6
0 to –40	357	10	367	2.7
0 to –45	603	38	641	5.9
0 to –50	804	120	924	13.0
0 to–∞	898	454	1352	33.6

^1^N target = number of expert-confirmed *V*. *chilensis* call events found in 26 manually tagged 14-min soundscapes.

See S4 Table for monthly values.

The recognizer output for each soundscape recording is a list of start/end timestamps of *V*. *chilensis* sound events, representing isolated single calls and call series. We point out that at the current stage of development we made no effort to build a recognizer that can distinguish between call series emitted by single birds, pairs, and groups of *V*. *chilensis*. Moreover we considered any split of call series into fragments undesirable. Thus, overlapping choruses emitted by different birds or groups of birds usually were merged within a single detection. Thereby we avoided that long call series were given more weight than isolated single calls and short call series. Otherwise the calling activity of floating nonbreeding lapwing flocks could have masked the expected increase in vocal activity during the main breeding period. Consequently, in the present study the term vocal activity refers to the cumulated number of detected sound events per time unit and does neither make a statement about the number of vocalizing lapwings nor about the number of call notes contained in each call series. We analyze the cumulated counts of *V*. *chilensis* acoustic events per hour, day, and month, as well as per daytime and nighttime.

### Validation datasets and assessment procedures

A bird sound expert prepared all training and validation datasets and assessed the detector results. Inspecting the raw detection results of the complete annual cycle of recordings (Table 1), we decided to focus our analysis on April to September 2013. The latter period includes the supposed main breeding season with the expected higher than average number of *V*. *chilensis* detections (*cf*. assumptions *A1-A4*).

We evaluated the *V*. *chilensis* recognizer performance through a two-pronged approach: (I) For the determination of the false positive rate we drew representative random samples from the *V*. *chilensis* detections computed for each month. (II) For all other performance statistics, we selected 26 soundscapes containing at least one correct positive detection. Detailed descriptions of the two validation datasets follow:

1. Random sample.—The minimum number of assessed detections was 150 for months with low activity levels and 2% of all detections for months with high acoustic activity of the target species (S1 Table). We implemented a very strict definition of false positives, counting as insertions even those sound events where the start/end timestamps referred to non-target species rather than to the faint *V*. *chilensis* signals discernible in the background of the same segment. In the case of doubts with respect to the identification of non-target bird species, we consulted xeno-canto (www.xeno-canto.org) and several comprehensive audio publications on Neotropical birds [68–70]. This way we could identify all but one non-target bird species that provoked detections. In a few cases, it remained unclear whether the detection was triggered by *V*. *chilensis* or by a non-target species, and as a result we excluded eight detections from the analysis. Subsequently we assessed the recognition results in terms of *precision* measured in percentages:
  $$Precision\;=\frac{H}{H+I}*100,\;\left[\%\right]$$ (1)
  where *H* (hits = true positives) indicates how many times the *V*. *chilensis* recognizer correctly detected a target vocalization and *I* stands for insertions (false positives). Here *H+I* is the valid sample of target and non-target sound events, i.e. the number of randomly selected detections minus the positives for which it remained unclear whether they were triggered by *V*. *chilensis* or by another species (S2 Table).
2. Validation library.—Determination of the total number of target events in soundscapes is a laborious task. For practical reasons, we took the following approach for selecting a proportionate subsample of soundscapes: (a) We considered only recordings for which the presence of *V*. *chilensis* sound events was confirmed on the basis of the aforementioned random sample. (b) For each month, we selected a minimum of four recordings, about one per week. (c) The target value of recognizer detections was 10 to 12 per recording, however we chose variant numbers of detections when no soundscapes were available in that range. (d) The target sample size of expert-confirmed *V*. *chilensis* sound events per month was ≥ 100 for the dB-level range 0 to –50 dB. (e) For September the sample size was only reached after annotating six soundscapes, resulting in a total of 26 tagged 14-min soundscapes for the entire study period (S3 Table).

For the manual tagging of the soundscapes we followed the approach of Ganchev et al. ([36]: section 2.1.3.3). Methodological details specific to the present work are outlined here: (A) For the purpose of manual tagging we made use of an FFT filter to reduce competing noises such as wind and insects by –100 dB in the frequency ranges 0–640 Hz and 12.6–24 kHz. (B) We annotated directly in Adobe Audition version 3.0 (www.adobe.com) by setting start and end time marker cues for each target event. (C) The recognizer-generated timestamps were not changed. Where various detections referred to different segments of the same call event, we regarded only the first detection as valid and subsequent detections as double hits (S3 Table). Likewise, we counted false positive detections separately. (D) For strong acoustic signals, we noted the compound dB-values of the loudest call (i.e. signal plus background) [*cf*. 36]. By contrast, for weaker target events below about –35 dB, we selectively filtered the background noise of the frequency bands between the harmonics of the loudest calls to avoid overestimation of the dB-values. (E) We repeated the manual screening for potential *V*. *chilensis* sound events and subsequent identification several times. Depending of the complexity of the soundscape, the time effort per 14-min sound file was between about 16 and 40 h.

For the performance assessment, we considered the following annotations for analysis (Table 2): (i) Confirmed target events, corresponding to the characteristic multi-harmonic type of lapwing vocalizations emitted by adults and immature birds (section The target species). (ii) Unconfirmed events, comprising audio signals that we could not identify with certainty as target or non-target, either because they were too faint or because background noise levels were too high.

We evaluated the recognition results in terms of four performance metrics: accuracy (Eq 2), correct (Eq 3), missed (Eq 4), and precision (Eq 1) measured in percentages (S1 Appendix):

$$Accuracy\;=\frac{H-I}{N}*100,\;\left[\%\right]$$ (2)

$$Correct\;=\frac{H}{N}*100,\;\left[\%\right]$$ (3)

$$Missed=\frac{N-H}{N}*100,\;\left[\%\right]$$ (4)

where *H* stands for hits, *I* for insertions, and *N* is the total number of confirmed target events according to the validation dataset. We calculated the *precision* (Eq 1) and *accuracy* (Eq 2) only for the sake of completeness, although it is evident that the number of annotated soundscapes was too small and not representative with respect to false positive detections when compared with the results of the random sample of detections.

### Acoustic activity patterns

**Fig 3:**
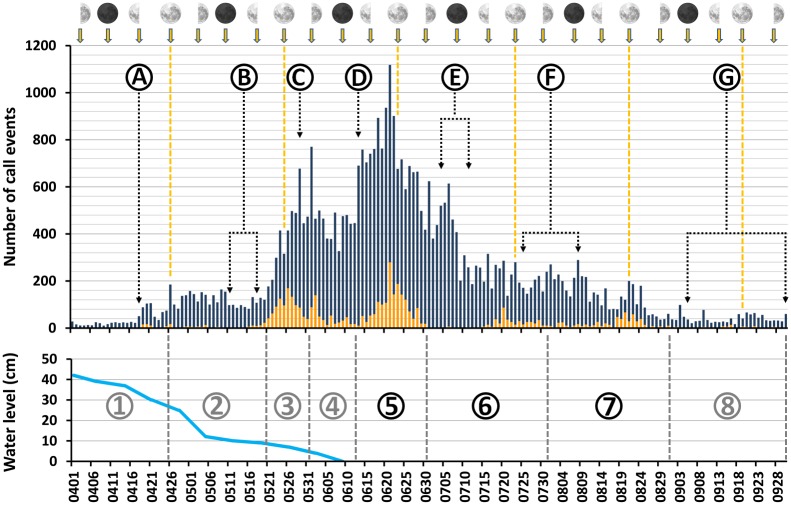
Corrected cumulated daily acoustic activity and variation of nocturnal activity of *Vanellus chilensis* in relation to lunar phases and water levels. Upper part: blue bars = diurnal activity; red bars = nocturnal activity; dashed orange lines = full moon nights. Black dotted lines mark key dates and periods in the *V*. *chilensis* life cycle, which in the text are referred to as *vcA*, *vcB*, etc.: A = occupancy of territory; B = egg-laying; C = day 12 of incubation; D = earliest date of hatching; E = change from downy to juvenile plumage; F = acquisition of flight (fledging), and G = attainment of independence by juveniles. Lower part: blue line = water level; dashed lines and circled numbers = periods for the calculation of average false positive rates, hereafter and in the main text referred to as *ap* for ‘acoustic activity period’; gray circled numbers = *ap* with false positive rates ≥ 10%; black circled numbers = *ap* with false positive rates < 10%. Original free image for the design of moon phase icons by courtesy of PhotoVaco.com (www.photovaco.com/photos/animals/full-moon-2-628).

**Fig 4:**
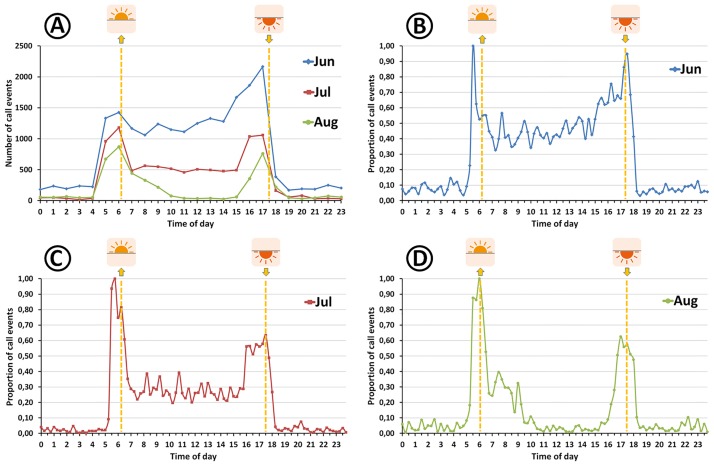
Cumulated diel acoustic activity of *Vanellus chilensis* in relation to sunrise and sunset in June (n = 20 498), July (9367), and August 2013 (4697). A: detections per hour for all three months; B-D: monthly proportion of detections per quarter hour. Hours of sunrise and sunset correspond to average values (dashed orange lines). Original public domain pictogram for the design of sunrise and sunset icons by courtesy of Icône.com (http://en.icône.com/tag-sun-0).

During the six months covering the presumed main breeding period, the false positive rates fluctuated widely between months (Table 1). In order to account for these pronounced changes, we split the study period into phases with similar error rates, calculated averages per period, and used the resulting percentage values to correct the number of daytime and nighttime detections (Table 3, Fig 3). Daytime detections included phases of increased activity around dawn and dusk, i.e. the entire period between one hour before sunrise and 45 min after sunset (Fig 4).

**Table 3:** Cumulated daytime and nighttime detections per acoustic activity period of *Vanellus chilensis* and calculation of precision.

Detection category	Acoustic activity period (no., mmdd)								Total
	1	2	3	4	5	6	7	8	
	0401–0425	0426–0520	0521–0531	0601–0612	0613–0630	0701–0731	0801–0831	0901–0930	
Raw detections daytime	2301	4093	4428	5796	12 355	8899	4078	1642	43 592
Raw detections nighttime	209	210	1281	607	1740	468	619	153	5287
Total raw detections	2510	4303	5709	6403	14 095	9367	4697	1795	48 879
N random sample, detections	109	124	117	107	293	200	150	150	1250
Proportion total detections (%)	4.3	2.9	2.0	1.7	2.1	2.1	3.2	8.4	2.6
N correct random detections	39	88	88	93	272	193	138	103	1014
N false random detections	70	36	26	13	19	6	11	47	228
N excluded random detections	0	0	3	1	2	1	1	0	8
N valid random detections	109	124	114	106	291	199	149	150	1242
*Precision* (%)	35.8	71.0	77.2	87.7	93.5	97.0	92.6	68.7	81.6
Corrected detections daytime	823	2905	3418	5085	11 548	8631	3777	1128	37 315
Corrected detections nighttime	75	149	989	533	1626	454	573	105	4504
Total corrected detections	898	3054	4407	5618	13 175	9085	4350	1233	41 819

See S1 Table for monthly values. The false positive rates (= 100 –precision [%]) were used to correct the number of diurnal and nocturnal detections (*cf*. Fig 3).

Uncorrected diurnal acoustic activity was analyzed only for those three months for which average false positive rates were below 10% (i.e. Jun.–Aug. 2013; Table 1), because for the remaining months unevenly distributed false positives may have resulted in distortions of the activity patterns. We plotted *per hour* and *per quarter-hour* views to demonstrate the effects of time resolution on the interpretation of diurnal activity patterns (Fig 4).

### Statistical tests

For testing the anticipated negative correlation between water level and *V*. *chilensis* vocal activity, we performed a one-tailed Spearman’s *rho* test, using the last ten water level measurements and the corresponding five-day averages of corrected lapwing detections. The water level reading from 1 April (= 42 cm) was omitted because with respect to the averages of acoustic activity this measurement lay outside the study period. Here we opted for a non-parametric correlation test because histogram plots revealed irregular distribution of the data.

Comparing the anticipated seasonal patterns (*cf*. assumptions *A1-A4* and section Duration of the breeding cycle) with Fig 3 we found seven life-cycle phases of *V*. *chilensis* acoustic activity for which we present means and standard deviations for small samples ($\bar{x}\;\pm s$).

Finally, to test for differences in variance of *V*. *chilensis* vocal activity between nights with and without moonlight we formed three samples, each containing 13 nights around (a) the new moon preceding the full moon phase to be tested, (b) the full moon phase itself, and (c) the next new moon. We excluded one or two nights around first quarter and last quarter moons, depending on the total length of the lunar phases. The data were log_10_-transformed before performing a one-way ANOVA (analysis of variance) with significance level at *p* = 0.05. Subsequently we used *post hoc* Tukey HSD (honestly significant difference) tests with significance level at *p* = 0.01 for pairwise multiple comparisons.

## Results

### Recognizer performance assessment

For the six-month period covering the supposed main breeding season, the automated sound recognizer detected 48 879 supposed target events (Table 1, S1 Table). Of these we validated the identification of 1250 detections, embedded in 1063 original soundscapes. The validation of the random sample of detections showed that the recognizer precision varied considerably during the breeding cycle (Table 3). Fourteen avian species triggered false positives, besides some other sound sources (S2 Table). The highest false positive rate was reached in April (52%), when *V*. *chilensis* vocal activity was particularly low. The lowest rates were found for the period June to August (3–8%) with high acoustic activity of the target species. In the period April to June the majority of misidentified audio signals were emitted by common non-passerine waterbirds, *viz*. Limpkin *Aramus guarauna* (n = 64), Wattled Jacana *Jacana jacana* (32), Black-bellied Whistling Duck *Dendrocygna autumnalis* (27), and White-backed Stilt *Himantopus melanurus* (10). By contrast, in the late dry season months July to September common landbirds triggered most false positives, namely Great Kiskadee *Pitangus sulphuratus* (20), Monk Parakeet *Myiopsitta monachus* (15), and Guira Cuckoo *Guira guira* (10).

**Fig 5:**
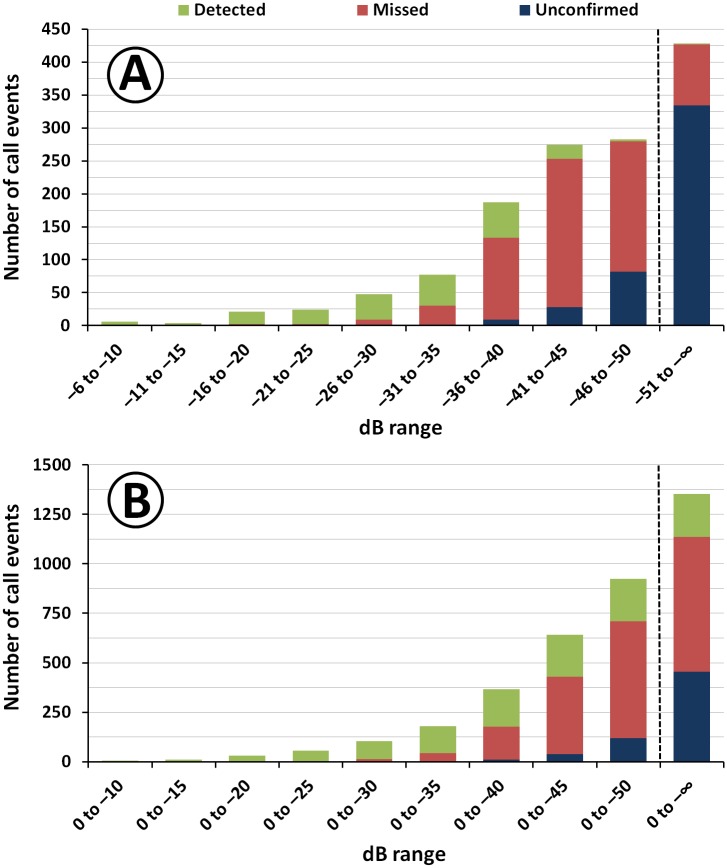
Recognizer performance, according to the loudest call amplitude: number of detected, missed, and unconfirmed *Vanellus chilensis* events in the validation library. A: per 5-dB category; B: cumulated number. The dashed lines indicate a change in scale. The ranges –51 to–∞ and 0 to–∞ dB show the effect of target vocalizations with an amplitude < –50 dB, which are barely identifiable even for a bird-sound expert.

The ratio between detected, missed, and unconfirmed target events demonstrates the limitations of the *V*. *chilensis* recognizer in comparison with human experts (Fig 5A and 5B). The percentage of correct hits rapidly decreased with attenuating signal strength, resulting in a rather low overall recognizer performance (S1 Appendix). However, monthly false negative rates in the amplitude range of 0 to –50 dB showed little variation at 72.7 ± 6.9%, with more misses in the period May to July (74.4–80.2%) and less in the other months (62.7–71.3%). Likewise, the amplitude of the weakest event detected per soundscape was rather invariable (–39.3 ± 5.4 dB, n = 26 out of 281 correct detections), with May (–35.3 dB) and June (–37.3 dB) being the months when signal amplitude had to be higher than in the other months to trigger detection (S3 Table). Unexpectedly, in a few recordings extremely weak target vocalizations were also detected, e.g. in July and August. Visual inspection of the corresponding audio files revealed that the recognizer had assigned positive hits to compound signals, consisting of loud cicada songs and faint target signals. The monthly rate of repeated detections of single *V*. *chilensis* call events was also rather steady (22.7–28.8%), with the exception of August when the rate was lower (13.8%; S3 Table).

### Seasonality of vocal activity

For the complete annual cycle of recordings the *V*. *chilensis* sound recognizer time-stamped a total of 62 292 call events (Table 1). As predicted by assumptions *A1* and *A3*, the number of lapwing detections fluctuated around an average value most of the year, i.e. 3000 ± 900 call events (Nov. 2012-Apr. 2013 and Aug.-Oct. 2013), and markedly increased above average levels only for a three-month period (May-Jul. 2013). The latter period was sufficiently long to cover the main *V*. *chilensis* breeding season [46,47]. The predicted decrease in acoustic activity during the height of the flood cycle (Feb.-Apr. 2013) was not discernible in the raw detection data (Table 1), likely due to the high activity of waterbirds triggering false positive detections. The 52% false positive rate found in the random sample of detections for April supports this interpretation (Table 1).

Based on seasonal shifts in the occurrence of sound emissions of *V*. *chilensis* and the non-target species we distinguished between eight different acoustic activity periods (S2 Table). Subsequently we determined period-specific false positive rates (Table 3) to correct the cumulated number of daily *V*. *chilensis* detections (Fig 3). This procedure barely affected the observed general pattern of seasonal acoustic activity (S4 Fig). In later sections below, prefix symbols for dates and periods given in parenthesis refer to those shown in Fig 3, termed hereafter *ap* for ‘acoustic activity period’, and *vc* for certain crucial events in the *V*. *chilensis* life cycle. All comments on the behavioral contexts of *V*. *chilensis* vocalizations (Fig 1A–1F) and the corresponding audio files provided in the Supporting Information (S1–S9 Audios) refer to sounds found in the random sample of 1250 detections.

### Daily activity patterns

Once we had accounted for erroneous detections, a one-tailed Spearman’s *rho* test confirmed a significant negative correlation between the height of the water level and the intensity of *V*. *chilensis* acoustic activity (*r*_*s*_ = –0.95, *p* < 0.004). As predicted by assumption *A2*, lapwing vocal activity was lowest in the first two weeks of April 2013, with 19 ± 6 call events detected per day (Fig 3: *ap*1). Correct detections of lapwings referred to birds vocalizing at a great distance to the microphone, resulting in faint signals. The behavior of *V*. *chilensis* began to change on 18 April when the water level fell to *c*. 30 cm and the species started to fly around recording station PPA001 (*vc*A). Thereafter lapwings patrolled the site regularly (Fig 1A and 1B, S1 and S2 Audios). Between 26 April and 20 May the water level dropped to 9 cm and acoustic activity stabilized around 110 ± 35 detections per day (*ap*2), with a slight decrease perceptible around mid-May (*vc*B). Subsequently, *V*. *chilensis* call frequency rose quickly from 21 May onwards (begin *ap*3) before it stabilized a few days later at a level of 467 ± 104 sound events per 24 h (end *ap*3 and *ap*4). Between 18 May and 12 June, we repeatedly noticed distinct short series of agitated alarm and cackling calls, indicative of adults at the nest or close to it (Fig 1C and 1D, S3–S7 Audios). During these weeks the floodwaters retreated completely (*cf*. S1A–S1C Fig). A sudden and sustained increase in *V*. *chilensis* activity was noted on 13 June and, based on assumption *A3*, interpreted as the earliest day of hatching (*vc*D). Thereafter daily detection rates fluctuated at 766 ± 133 hits for about two weeks (*ap*5) with a maximum value of 1117 call events reached on 21 June. Most detections in that period referred to extensive alarm call series of adults mobbing potential predators and food competitors in defense of their chicks (Fig 1E and 1F, S8 and S9 Audios). On 29 June vocal activity fell again to about 489 ± 84 detections per day (end *ap*5 and begin *ap*6; *vc*E). A pronounced drop in daily activity occurred on 9 July (end *vc*E). During the subsequent seven weeks, vocal activity slowly decreased further, with averages for the entire period varying at 220 ± 50 events per day (*ap*6 and *ap*7). Finally, in September *V*. *chilensis* acoustic activity was almost as low as in early April, with daily detections of 43 ± 18 (*ap*8 and *vc*G).

During the three lunar phases from June to August, *V*. *chilensis* nocturnal acoustic activity was higher in moonlight nights when compared with nights around the new moon (Fig 3; ANOVA, *F*_2,36_ = 22.9 to *F*_2,36_ = 116.9, *p* < 0.001). However, a *post hoc* Tukey HSD showed that at the end of the breeding period differences in nocturnal activity means were not significant for the new moon and full moon phases in August, possibly because of the recording gap in the night from 17 to 18 August.

### Diel activity patterns

Diurnal vocal activity was analyzed in two time resolutions, showing that *per-hour* sums of detections (Fig 4A) mask many interesting details discernible in *per-quarter-hour* views (Fig 4B–4D). Generally speaking, *V*. *chilensis* acoustic activity peaked around dawn and dusk. However in the *per-quarter-hour* projections, but not in the *per-hour* view, it is perceptible that vocal activity varied in time even between the activity peaks at dawn and dusk; that is, activity tended to peak periodically every 45 to 120 min.

The most striking difference in diurnal activity patterns between months is the high level of daytime activity in June, when detection rates fluctuated around 45% of maximum activity even during midday heat. Furthermore, in the afternoon a sustained increase of activity occurred as early as 2.5 h before sunset. According to assumption *A4*, we regard an all-day-active pattern as indicative of lapwings tending chicks. The situation was similar but less pronounced in July, when the proportion of call events decreased to 30% of maximum activity in the late morning hours and to 25% in the early afternoon. Likewise, the strong increase of afternoon activity began about 45 min later than in June. By contrast, in August *V*. *chilensis* daytime activity dropped to less than 5% between 10:00 and 15:30 h and a strong increase in afternoon activity set in only about 1.5 h before sunset.

## Discussion

### How well do the recognizer results reflect the real vocal activity patterns of *Vanellus chilensis*?

With the help of two validation datasets, we assessed the effects of five potential sources of bias, which may have had some modulation effect on the determination of time-varying *V*. *chilensis* call rates. Here we explain why we think that the identified biases had only a moderate effect on the final results. (1) Time-varying false positive rate (Table 3).—The general vocal activity pattern was not affected by the *post-hoc* correction of recognizer results, despite the pronounced fluctuation in monthly false positive rates (*cf*. Fig 3 and S4 Fig). (2) High false negative rate (S1 Appendix, Fig 5A and 5B).—For the amplitude range of 0 to –50 dB, the monthly false negative rates showed only little variation, particularly for the months of highest activity (May-Jul.). Even in the range of 0 to –40 dB, where time variation was evident and most target signals were detected, miss rates were much lower for months with low *V*. *chilensis* activity, April (22.7%) and September (27.9%), than for the high-activity months May-July (50.8–63.6%). August (40.8%) was intermediate. (3) Varying recognizer sensitivity (S3 Table).—The signal amplitude of the weakest target event detected in soundscapes was rather stable. Moreover, in May and June call events had to be stronger than in other months in order to trigger detections. (4) Unexpected detection of very weak target signals.—In two soundscapes in the validation library, the recognizer detected very weak *V*. *chilensis* signals in the presence of loud and monotonous cicada songs (S3 Table). Similarly, in the random sample we found eight ambiguous cases where low-amplitude target events were detected in combination with stronger signals from competing bird and frog species (S2 Table). Less ambiguous cases of combined detections were either counted as correct hits or false positives (section Validation dataset and assessment procedures). The most probable reason for such combined detections is the training library, which despite of manual filtering still contained some faint audio signals of competing species. However, considering the small number of combined detections found in the random sample, it is unlikely that they had a strong effect on the final detection results. (5) Repeated detection of different segments of a single call event (S3 Table).—The double-hit rate was almost constant, making it improbable that repeated detection of single call events had a strong effect on detection results. We interpret the lower rate in August as an outlier due to small sample size.

In summary, all factors analyzed here indicate that in the high-activity period (May-Jul.) the number of detected target events was rather underestimated than overestimated when compared with the low-activity months (Apr., Aug., and Sep.). Thus the recognizer results provide a good approximation of the general *V*. *chilensis* vocal activity patterns, although we did not investigate whether this also holds true for short-term changes like day-by-day variability (*cf*. Fig 3). Most importantly for the present work, the corrected detections fit the acoustic activity patterns expected to be found during the main breeding season rather well (*cf*. assumptions *A1-A4* and section Duration of the breeding cycle). The other data discussed in the subsequent sections provide additional support for our interpretations, namely water level (Fig 3), periodic occurrence of distinct *V*. *chilensis* vocalization types (S1–S9 Audios), seasonal abundance shifts in (sound-producing) insect species (S10–S12 Audios), and published references on the reproductive strategies of other bird species.

### *Vanellus chilensis* life-cycle phases

In the Venezuelan Llanos, a seasonally-flooded savannah very similar to the northern Pantanal, the main breeding period falls in the early rainy season, April-July ([46]: n = 17 nests). Likewise in Central Brazil’s *Planalto* region *V*. *chilensis* breeds in the late dry and early rainy season, July-December ([51]: n = 98 breeding attempts; [50]: n = 74 nests). By contrast, Amazonian populations of charadriids migrate in response to the seasonal inundation of their floodplain habitats and initiate breeding as soon as the waters recede ([54]: p. 222). Our data suggest that in the northern Pantanal *V*. *chilensis* shows a seasonality pattern similar to Amazonian populations of charadriids, probably because flooding is extensive in our study area (S1 and S2 Figs). As predicted by assumption *A2*, we demonstrate that for the six-month period analyzed here, calling frequency was lowest in the first half of April 2013 when the water level still was >30 cm (Fig 3). However, at the end of the flood cycle the monthly number of *V*. *chilensis* detections at PPA001 sharply increased (*cf*. assumption *A3*). The maximum of 18 151 raw detections in June was about six times higher than the nine-months average value of 3000 ± 900 of the low activity period (*cf*. assumptions *A1* and *A3*; Table 1). Based on the manually inspected random sample of detections we exclude the possibility that the increase was due to the presence of nonbreeding flocks (*cf*. S1–S9 Audios; details given in the next sections). In June and July we also noted high levels of parental care behavior and dial vocal activity patterns showed the characteristic all-day active mode (Fig 4B and 4C), predicted by assumption *A4* for lapwings tending young [47,48]. In August behavioral patterns already had reverted to nonbreeding mode (Fig 4D).

#### Territory establishment

*Vanellus chilensis* is philopatric with respect to its breeding territories [50,51]. However in early April 2013, when the floodwater receded from its February/March maximum of *c*. 65 cm (S2 Fig), some lapwings were already present in the Fazenda Pouso Alegre area (Fig 3: *ap*1). In concordance with assumption *A2*, most lapwings vocalized at a substantial distance from PPA001. Hitherto the floodwaters had disappeared only from some higher areas, such as certain dirt roads and cattle gathering sites, approximately 300 to 500 m from the recorder (*cf*. S1A Fig, showing the extent of flooding on 4 May).

In agreement with Walters & Walters [46] our data confirm that lapwings reoccupied their territories even before the floodwaters completely receded (S1A Fig, Fig 1A and 1B; Fig 3: *vc*A, S1 and S2 Audios). Moreover, the low nocturnal activity of *V*. *chilensis* during moonlight nights in late April suggests that the lapwings roosted in one of the distant *terra firma* areas rather than on their inundated territories.

According to the literature mean territory size of *V*. *c*. *lampronotus* is 3–4 ha, independent of group size [51], corresponding to an area of up to 200 x 200 m. Admittedly our experimental setup does not permit the determination of population densities, but in some audio recordings we can distinguish at least 3 to 4 dispersed lapwing RSUs vocalizing simultaneously at various distances from the microphones. At least one pair or group bred close to station PPA001 and we presume that these birds emitted a high proportion of the valid detections.

#### Egg laying and incubation

Based on assumption *A3*, we conclude that the date of earliest hatching was the 13 June (Fig 3: *vc*D) because average daily activity suddenly increased by 64% to maximum levels (*cf*. *ap3* and *ap4* with *ap5*). The calling intensity remained very high for about 16 days. Thus, calculating with laying intervals of *c*. 2 days, an average clutch size of 3 eggs [46,50], and an incubation period of 26–27 days [46,52,66], we roughly calculate that egg-laying at PPA001 took place in the period 11–18 May (*vc*B). By then the water level had fallen to *c*. 10 cm and the first ‘islands’ appeared on the drying plain (S1A and S1B Fig). This period coincided with a slight decrease in lapwing vocal activity. Interestingly, Brunton ([71]: Fig 5) also reported very low levels of parental defense behavior during the initial phase of a breeding attempt in another precocial charadriid, the Killdeer *Charadrius vociferus*. In our study area, egg-laying before 6 May seems rather unlikely due to the much higher water level in the days before (Fig 3). Likewise, the gradual increase in vocal activity starting on 21 May is most consistent with the expected increase in defense activity of incubating lapwings [47–49,52], making a later incubation start implausible. During that period (*ap*3 and *ap*4), lapwings regularly emitted vocalizations typical for incubating birds (Fig 1C and 1D, S3–S7 Audios). By contrast, call series given by birds and flocks mobbing predators and interspecific competitors sound very different (Fig 1E and 1F, S8 and S9 Audios).

Incubating lapwings reduce the time spent on activities away from the nest to a minimum [47,48]. However, active defense of the clutch may not be immediate; for instance, Naranjo [52] reported agonistic behavior by both adults only from day 12 of incubation. By contrast, we noted increased calling activity within the first week of the presumed start of incubation and by day 12 (i.e. 29 May = *vc*C) calling frequency had increased to an average of *c*. 470 per day. Vocal activity levels remained high for almost three weeks before jumping to the maximum level between 13 and 28 June. We regard the high species richness and population density of potential predators in the Pantanal wetlands as the likely reason for the early increase in *V*. *chilensis* calling activity, since adult lapwings are forced to regularly defend themselves and especially their clutch against predators [49].

#### Parental care

Antipredator response in Southern Lapwings is precisely adapted to the behavior of the predator and the stage of the breeding cycle and is most intense in adults tending small chicks (*cf*. assumption *A3*). Brunton [71] found a similar behavioral pattern for the Killdeer *Charadrius vociferus*. Defense intensity and risk taking (*cf*. [71]: Fig 5) increased from early to late incubation. Parental defense behavior in *C*. *vociferus* reached maximum levels two days before hatching and continued at high level for another 4–5 days. Eventually defense activity declined quite quickly, presumably because in precocial species offspring vulnerability peaks at hatching due to increasing mobility of older chicks [53]. However, in contrast to the more passively tending Killdeer ([72], *cf*. [48]: p. 265), Southern Lapwings are active tenders. Both sexes alternate in deliberately following, leading, and gathering their chicks, while the other mate is “off duty” foraging away from the young [47,48].

After the presumed date of earliest hatching on 13 June (Fig 3: *vc*D) acoustic activity continued to increase until 21 June, which we regard to be the latest possible hatching date. However it is likely that the latter increase was due to the extended light availability around full moon (Fig 3). The intense parental care of lapwings tending young effectively means that diurnal activity patterns switch from the nonbreeding two-peak mode, with high activity concentrated around dawn and dusk and very low activity levels during the midday heat ([55]; Fig 4D), to an all-day-active mode ([48]; Fig 4B and 4C; *cf*. assumption *A4*). Prompt reversal to the nonbreeding mode of diurnal activity in August (Fig 4D), when young presumably started to fly, is a strong indication that continued daytime activity is costly for adults. Besides the reduced time available for foraging, there is the serious problem of thermoregulation during midday heat [47,48] and an increased risk of adults falling victim to predators when defending young [61]. By contrast, lapwings breeding in groups spend more time foraging than adults of pairs without helpers [47,48]. Not surprisingly, disadvantages of parental care are potential evolutionary drivers for the development of cooperative breeding systems in *V*. *chilensis*, beyond the evident benefits for chick survival [46,50].

That reproductive effort is indeed costly for adults, even in rather large and long-lived bird species, has been exemplarily demonstrated for the California Gull *Larus californicus* [73–75]. Older *L*. *californicus* parents invest more effort in feeding and defense of chicks than younger pairs and these higher levels of parental care by older birds are associated with higher adult mortality.

Strangely, when chicks were presumed to change from downy to juvenile plumage we noted a pronounced decrease in acoustic activity (*vc*E). It is unclear whether this drop in activity levels was caused by the loss of chicks or because juvenile-plumaged birds are better at recognizing and hiding from predators than downy chicks. Considering that diurnal activity patterns remained in breeding mode in July (Fig 4C), it is likely that at least one young fledged successfully (*vc*F). However, our results may comprise the vocal activity of more than one RSU, so that the activity pattern may indeed reflect successful reproduction in neighboring territories rather than of the lapwings breeding closest to PPA001. We anticipate that a closer inspection of the detected call events will reveal a clearer picture in this respect.

#### Post-breeding behavior

After the breeding season the formation of flocks allows adults and juveniles alike to optimize their time budgets and to save energy [47,48]. During morning hours lapwings forage singly or in small groups, whereas during the midday heat group size increases and less demanding activities, like preening, loafing, and roosting prevail [55]. Finally, in the afternoon the decrease in group size is accompanied by an increase in general activity. Overall, this behavioral pattern explains the nonbreeding mode of diurnal *V*. *chilensis* vocal activity in our study area very well (Fig 4D).

**Fig 6:**
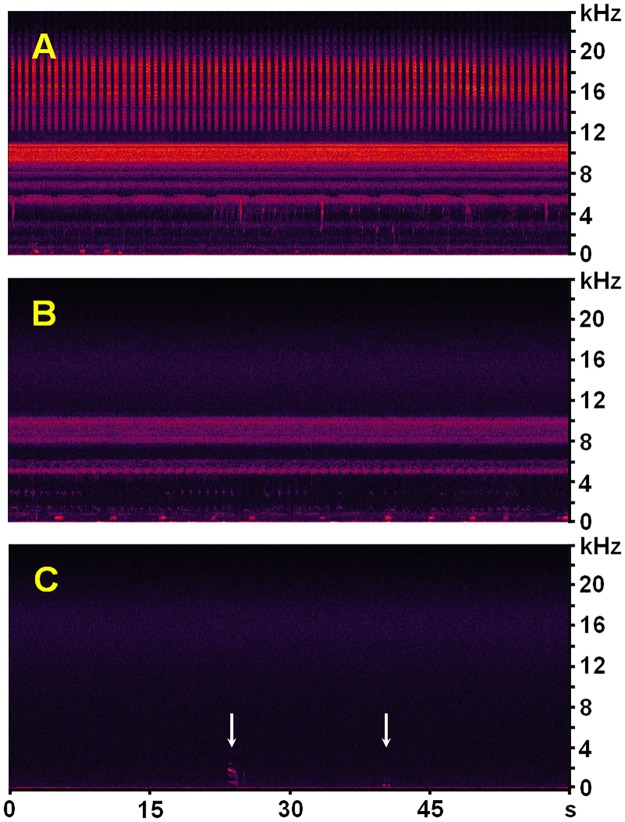
Spectrograms of exemplary midnight soundscapes. All spectrograms represent unfiltered 60-s segments of WAV soundscapes passively recorded at station PPA001 in the Fazenda Pouso Alegre area (minutes 5:30–6:30 of the original 14-min soundscapes; windowing function: Blackman-Harris; resolution: 512 bands; decibel range: 85 dB; amplification = +10 dB; *cf*. S10–S12 Audios). A: at the height of the rainy season several species of sound-producing insects, anurans, birds, and mammals (mostly cattle) fill the entire frequency range 0–22 kHz with audio signals; 25 Jan. 2013, 23:49 h (S10 Audio). B: at the end of the rainy season when *V*. *chilensis* reestablished its territories, sound-producing insects and anurans are still present in the frequency range 0–10.5 kHz; in addition, some of the signals below 4 kHz were emitted by birds and Zebus *Bos indicus*; 25 Apr. 2013, 23:54 h (S11 Audio). C: at the height of the dry season sound-producing insects and anurans are virtually absent in most soundscapes. The white arrows mark the faint signals of a Feral Horse *Equus ferus caballus* (left) and a Zebu (right); 25 Jul. 2013, 23:51 h (S12 Audio).

One striking finding was that *V*. *chilensis* had already abandoned the area of PPA001 less than two months after the floodwaters receded (S1A–S1F Fig; Fig 3: *vc*G), most likely because of a food shortage (*cf*. [46]: p. 508]). Lapwings feed on small invertebrates, such as earthworms, mollusks, spiders, woodlice, hemipterans, ground beetles, weevils, grasshoppers, and hymenopterans, as well as larvae of lepidopterans, coleopterans, and dipterans, using a run-and-pause technique that includes quick pecks and shallow probing on land [44,47,66,76–78] and sometimes foot-trembling in shallow water [45,54]. During the dry season, grassland-inhabiting invertebrates become less accessible to the birds. Declines in invertebrate populations have been described for several tropical ecosystems with a pronounced seasonal climate, such as savannahs [79] and *Cerrado*, e.g. earthworms migrate to deeper soil layers, enter a diapause, or are completely absent [80,81] and snails go into estivation [82]. Certain arthropods are sensitive to drought as well, including some of the groups known to be crucial food resources for lapwings [83,84]. Sound-producing insects, like orthopterans and cicadas, are conspicuously evident in our wet season soundscapes (Fig 6A and 6B, S10 and S11 Audios). By contrast, dry season recordings are usually very clean in the entire frequency range between 0–24 kHz, indicating a possible absence of most sound-emitting insect species or at least of their imago stages (Fig 6C, S12 Audio).

#### Nocturnal activity

Although nocturnal foraging is common in several wader species [85], charadriids are obligate visual foragers [86,87]. Our results demonstrate that increased nighttime *V*. *chilensis* activity correlates with available moonlight (Fig 3). This finding is consistent with the physiologically enhanced night vision capabilities of plovers [88]. In urban and industrial areas *V*. *chilensis* largely benefits from artificial illumination [58].

#### Concluding remarks on the breeding cycle

Based on four assumptions (*A1-A4*) about the seasonality of vocal activity we demonstrate that the 2013 breeding period of *V*. *chilensis* fell in a narrow time window between the end of the flood cycle and the height of dry season (May-Jul.). The raw detection data of the complete 2012/2013 annual cycle of recordings provide no indication that lapwings reproduced outside the main breeding season (Table 1). One consequence of this timing is that the species reproduced several months earlier than *V*. *c*. *lampronotus* populations in Central Brazil’s *Planalto* region [50,51]. Furthermore, the food shortage that occurred only two months after the end of the flood cycle makes it rather unlikely that *V*. *chilensis* routinely breeds more than once in our study area. This does not exclude the possibility that the species lays another clutch if breeding attempts fail [46,47,51].

Considering that in the northern Pantanal local topography causes floodwaters to rise and recede asynchronously in different areas, we presume that lapwings breed in other months at locations less prone to inundation than our study area. Likewise, we expect year-to-year differences caused by annual variations in the extend and timing of flooding.

### Comparison with published research

Previous studies based on automated recognition have presented diurnal and seasonal acoustic activity patterns of insects [18], anurans [17,18,43], birds [18,19], and mammals [18,42]. However, with exception of Frommolt and Tauchert [19], who presented quarter-hour sums for the nocturnal booming activity of Eurasian Bittern *Botaurus stellaris*, the time resolution of comparable studies has been low, i.e. diel activity was presented as cumulated hourly detection rates, which may mask interesting details of the calling activity (Fig 4A–4D). Similarly, most studies on seasonal changes in acoustic activity have only presented monthly detection frequencies [17,18,42,43], which conceal short-term activity changes. Therefore none of the latter studies has linked calling activity levels to multiple life-cycle phases of the studied animals. Here we explore this untapped potential of automated acoustic monitoring. The data obtained by means of automated sound event recognition permit the characterization of *V*. *chilensis* diurnal and seasonal activity patterns in unprecedented detail. Most strikingly, on the basis of pronounced shifts in the intensity of adult acoustic activity, it seems feasible to determine the start and end of each *V*. *chilensis* life cycle phase with presumed error margins of only a few days. We anticipate that passive sound recording combined with field observations will make interpretations of automatically detected acoustic activity even more precise.

### Follow-up research

Promising research directions for improving the overall recognition accuracy on field recordings are audio parameterization methods that make use of image processing techniques applied on the spectrogram seen as an image [18,34,89–95]. The consideration of domain knowledge about the structure of the species-specific sound events and the acoustic model of the operational environment in the classification step are other options to improve recognizer performance [36]. Finally, rule- and grammar-based methods for post-processing the output of the log-likelihood ratio estimator could allow calibrating the detector specifically for distinct *V*. *chilensis* vocalization types represented in the training library. However at present the availability of training, background, and validation libraries is the principal bottleneck in the development of species-specific and vocalization-specific acoustic recognizers. The compilation of comprehensive high-quality sound libraries is demanding. Only experienced bioacousticians, familiar with the acoustic vocabulary of the target and other sound-producing species, are qualified to build adequate datasets.

## Supporting Information

S1 Appendix Monthly and cumulated recognizer performance according to the loudest call amplitude in *Vanellus chilensis* vocalizations. (PDF)

S1 Audio Flight calls of *Vanellus chilensis* patrolling its inundated territory in the Fazenda Pouso Alegre area. Background (faint): a second Southern Lapwing, frogs, and insects. Recording station PPA001, 28 Apr. 2013, 17:38 h; unfiltered (Fig 1A).
(MP3)

S2 Audio Display, including agitated cackling, of a *V*. *chilensis* pair flying over their inundated territory. Recording station PPA001, 29 Apr. 2013, 16:35 h; gradually filtered in the range 0–0.5 kHz to remove wind noise (Fig 1B).
(MP3)

S3 Audio Single-note alarm calls and agitated cackling of a *V*. *chilensis* pair, presumably close to their nest. Background (partially faint): another Southern Lapwing, Snowy Egret *Egretta thula* (three loud calls), Bare-faced Ibis *Phimosus infuscatus*, Black-bellied Whistling Duck *Dendrocygna autumnalis*, and insects. Recording station PPA001, 26 May 2013, 08:09 h; gradually filtered in the range 0–0.5 and 8.4–9.2 kHz to reduce wind and insect noise respectively.
(MP3)

S4 Audio Example 1 for short series of cackling alarm calls of perched *V*. *chilensis* close to its nest. Background (faint): a second Southern Lapwing, *E*. *thula*, Yellow-chevroned Parakeet *Brotogeris chiriri*, and insects. Recording station PPA001, 31 May 2013, 14:31 h; unfiltered (Fig 1C).
(MP3)

S5 Audio Example 2 for short series of cackling alarm calls of perched *V*. *chilensis* close to its nest. Background (faint): Southern Lapwing and other bird species. Recording station PPA001, 11 Jun. 2013, 14:21 h; unfiltered.
(MP3)

S6 Audio Example 1 for short series of agitated alarm calls of *V*. *chilensis* flushed from its nest or close to it. Background (faint): *D*. *autumnalis*, Greater Rhea *Rhea americana* (low frequency boom), and others. Recording station PPA001, 30 May 2013, 06:13 h; unfiltered (Fig 1D).
(MP3)

S7 Audio Example 2 for short series of agitated alarm calls of *V*. *chilensis* flushed from its nest or close to it. Background (faint): other Southern Lapwings, *D*. *autumnalis*, and others. Recording station PPA001, 06 Jun. 2013, 14:15 h; unfiltered.
(MP3)

S8 Audio Example 1 for long alarm call series of a *V*. *chilensis* flock mobbing a predator (e.g. Southern Crested Caracara *Caracara plancus*) to defend their chicks. Background (faint): *E*. *thula*, *C*. *plancus* (faint cackling), other birds, and insects. Recording station PPA001, 16 Jun. 2013, 15:35 h; filtered in range 0–100 Hz to remove wind noise (Fig 1E).
(MP3)

S9 Audio Example 2 for long alarm call series of a *V*. *chilensis* pair or flock mobbing a potential predator or food competitor (e.g. Limpkin *Aramus guarauna*) to defend their chicks. Background (partially faint): *A*. *guarauna* (faint cackling), *E*. *thula*, Yellowish Pipit *Anthus lutescens*, other birds, and wind. Recording station PPA001, 21 Jun. 2013, 12:28 h; filtered in range 0–150 Hz to remove wind noise (Fig 1F).
(MP3)

S10 Audio Midnight soundscape at the height of the rainy season in the Fazenda Pouso Alegre area. Sound-producing insects and anurans, at least 6 species each, fill the entire frequency range 0–22 kHz with audio signals. Other species (mostly faint): White-faced Whistling Duck *Dendrocygna viduata*, *D*. *autumnalis*, Wattled Jacana *Jacana jacana*, Rufescent Tiger-Heron *Tigrisoma lineatum*, Zebu *Bos indicus*, and others. Recording station PPA001, 25 Jan. 2013, 23:49 h; unfiltered, loudest signal equalized to –6 dB (Fig 6A).
(MP3)

S11 Audio Midnight soundscape at the end of the rainy season when *V*. *chilensis* reestablished its territories in the Fazenda Pouso Alegre area. Sound-producing insects and anurans, at least 2–3 species each, are still dominant in the frequency range 0–10.5 kHz. Other species (mostly faint): *D*. *viduata*, *D*. *autumnalis*, Cocoi Heron *Ardea cocoi*, Black-crowned Night Heron *Nycticorax nycticorax*, *A*. *guarauna*, *V*. *chilensis*, *B*. *indicus*, and others. Recording station PPA001, 25 Apr. 2013, 23:54 h; unfiltered, loudest signal equalized to –6 dB (Fig 6B).
(MP3)

S12 Audio Midnight soundscape at the height of the dry season. Sound-producing insects and anurans are virtually absent. Background species (faint): *R*. *americana*, *N*. *nycticorax*, *B*. *indicus*, Feral Horse *Equus ferus caballus*, and others. Recording station PPA001, 25 Jul. 2013, 23:51 h; unfiltered, loudest signal equalized to –9 dB (Fig 6C).
(MP3)

S1 Fig Fazenda Pouso Alegre area in the northern Pantanal, municipality of Poconé, Mato Grosso, Brazil, during the *Vanellus chilensis* breeding season in 2013. Landsat images by courtesy of the U.S. Geological Survey (http://landsatlook.usgs.gov): A = 4 May, B = 5 Jun., C = 7 Jul., D = 8 Aug., E = 24 Aug., and F = 25 Sep.; in 2013 no cloud-free images were available before and after these dates, including the period of maximum water levels in February/March 2013.
(PDF)

S2 Fig *Vanellus chilensis* habitat near recording station PPA001 in the Fazenda Pouso Alegre area. A: flooded cattle pasture with macrophytes at the height of the flood season. The water level at PPA001 was about 65 cm on that date (24 Feb. 2013). B: dry savannah-like cattle pasture at PPA001 at the end of the *V*. *chilensis* breeding season (2 Aug. 2013).
(PDF)

S3 Fig *Vanellus chilensis lampronotus* searching for food at a muddy lake margin in the Fazenda Pouso Alegre area (18 Jul. 2012). (PDF)

S4 Fig Uncorrected *Vanellus chilensis* cumulative daily acoustic activity for the period April to September 2013. *Cf*. Fig 3, Table 3, and S2 Table.
(PDF)

S1 Table Cumulated monthly daytime and nighttime detections of *Vanellus chilensis* call events and calculation of precision for the period April to September 2013. See Table 3 for acoustic activity periods.
(PDF)

S2 Table Identified sound sources in a random sample of 1250 detections, drawn from 48 879 *Vanellus chilensis* recognizer detections in soundscapes recorded at monitoring station PPA001, Pantanal region, Brazil, between April and September 2013. The false positive rates (= 100 –*Precision* [%]) were used to correct the number of diurnal and nocturnal detections in Fig 3.
(PDF)

S3 Table Validation library: soundscapes annotated for the performance assessment of the *Vanellus chilensis* recognizer. See S1 Appendix for performance assessment results. Double hits refer to repeated detections of the same lapwing call series and were omitted in subsequent analyses. The weakest event in dB refers to the loudest note of the weakest *V*. *chilensis* call event detected by the automated recognizer among correct hits and double hits.
(PDF)

S4 Table Validation Library: monthly number of confirmed and unconfirmed sound events according to the loudest call amplitude in *Vanellus chilensis* vocalizations. Table 2 presents the cumulated numbers (Apr. to Sep. 2013).
(PDF)
