# Supplementary material for: Automated Sound Recognition Provides Insights into the Behavioral Ecology of a Tropical Bird
Source: PLoS One. 2017 Jan 13;12(1):e0169041. doi: 10.1371/journal.pone.0169041 (PMC5235375; doi:10.1371/journal.pone.0169041)
Supplement: S1 Appendix — (PDF) [file pone.0169041.s001.pdf]

S1 Appendix. Monthly and cumulated recognizer performance according to the loudest call amplitude in *Vanellus chilensis* vocalizations.

		**dB range**										
**Period**	**Statistics**	**0 to –5**	**0 to –10**	**0 to –15**	**0 to –20**	**0 to –25**	**0 to –30**	**0 to –35**	**0 to –40**	**0 to –45**	**0 to –50**	**0 to –∞**
April	Hits, *H*	0	0	0	3	6	12	18	34	35	35	35
	Misses, *N–H*	0	0	0	0	0	0	1	10	49	87	100
	Insertions, *I*	0	0	0	0	0	0	0	0	0	0	0
	Total instances, *N*	0	0	0	3	6	12	19	44	84	122	135
	Accuracy	NA	NA	NA	100.0	100.0	100.0	94.7	77.3	41.7	28.7	25.9
	Correct	NA	NA	NA	100.0	100.0	100.0	94.7	77.3	41.7	28.7	25.9
	Missed	NA	NA	NA	0.0	0.0	0.0	5.3	22.7	58.3	71.3	74.1
	Precision	NA	NA	NA	100.0	100.0	100.0	100.0	100.0	100.0	100.0	100.0
May	Hits, *H*	0	2	3	5	11	14	21	24	25	25	25
	Misses, *N–H*	0	0	0	0	2	5	13	37	77	100	113
	Insertions, *I*	0	0	1	2	3	5	6	6	6	6	6
	Total instances, *N*	0	2	3	5	13	19	34	61	102	125	138
	Accuracy	NA	100.0	66.7	60.0	61.5	47.4	44.1	29.5	18.6	15.2	13.8
	Correct	NA	100.0	100.0	100.0	84.6	73.7	61.8	39.3	24.5	20.0	18.1
	Missed	NA	0.0	0.0	0.0	15.4	26.3	38.2	60.7	75.5	80.0	81.9
	Precision	NA	100.0	75.0	71.4	78.6	73.7	77.8	80.0	80.6	80.6	80.6
June	Hits, *H*	0	2	2	6	9	16	27	32	34	34	34
	Misses, *N–H*	0	0	0	1	1	5	11	33	73	99	114
	Insertions, *I*	0	0	0	1	1	1	1	1	1	1	1
	Total instances, *N*	0	2	2	7	10	21	38	65	107	133	148
	Accuracy	NA	100.0	100.0	71.4	80.0	71.4	68.4	47.7	30.8	24.8	22.3
	Correct	NA	100.0	100.0	85.7	90.0	76.2	71.1	49.2	31.8	25.6	23.0
	Missed	NA	0.0	0.0	14.3	10.0	23.8	28.9	50.8	68.2	74.4	77.0
	Precision	NA	100.0	100.0	85.7	90.0	94.1	96.4	97.0	97.1	97.1	97.1

S1 Appendix. Continued.

Period	Statistics	dB range										
		**0 to –5**	**0 to –10**	**0 to –15**	**0 to –20**	**0 to –25**	**0 to –30**	**0 to –35**	**0 to –40**	**0 to –45**	**0 to –50**	**0 to –∞**
July	Hits, *H*	0	0	1	2	5	11	20	28	34	35	35
	Misses, *N*–*H*	0	0	0	0	0	1	11	49	87	142	162
	Insertions, *I*	0	0	0	0	0	0	0	0	0	0	0
	Total instances, *N*	0	0	1	2	5	12	31	77	121	177	197
	Accuracy	NA	NA	100.0	100.0	100.0	91.7	64.5	36.4	28.1	19.8	17.8
	Correct	NA	NA	100.0	100.0	100.0	91.7	64.5	36.4	28.1	19.8	17.8
	Missed	NA	NA	0.0	0.0	0.0	8.3	35.5	63.6	71.9	80.2	82.2
	Precision	NA	NA	100.0	100.0	100.0	100.0	100.0	100.0	100.0	100.0	100.0
August	Hits, *H*	0	0	0	2	4	9	18	29	39	39	39
	Misses, *N*–*H*	0	0	0	1	1	2	3	20	58	82	96
	Insertions, *I*	0	0	0	0	0	0	0	0	0	0	0
	Total instances, *N*	0	0	0	3	5	11	21	49	97	121	135
	Accuracy	NA	NA	NA	66.7	80.0	81.8	85.7	59.2	40.2	32.2	28.9
	Correct	NA	NA	NA	66.7	80.0	81.8	85.7	59.2	40.2	32.2	28.9
	Missed	NA	NA	NA	33.3	20.0	18.2	14.3	40.8	59.8	67.8	71.1
	Precision	NA	NA	NA	100.0	100.0	100.0	100.0	100.0	100.0	100.0	100.0
September	Hits, *H*	0	2	4	11	16	28	33	44	45	47	48
	Misses, *N*–*H*	0	0	0	0	0	0	3	17	47	79	97
	Insertions, *I*	0	0	0	1	1	2	2	2	2	2	2
	Total instances, *N*	0	2	4	11	16	28	36	61	92	126	145
	Accuracy	NA	100.0	100.0	90.9	93.8	92.9	86.1	68.9	46.7	35.7	31.7
	Correct	NA	100.0	100.0	100.0	100.0	100.0	91.7	72.1	48.9	37.3	33.1
	Missed	NA	0.0	0.0	0.0	0.0	0.0	8.3	27.9	51.1	62.7	66.9
	Precision	NA	100.0	100.0	91.7	94.1	93.3	94.3	95.7	95.7	95.9	96.0

S1 Appendix. Continued.

		**dB range**										
**Period**	**Statistics**	**0 to –5**	**0 to –10**	**0 to –15**	**0 to –20**	**0 to –25**	**0 to –30**	**0 to –35**	**0 to –40**	**0 to –45**	**0 to –50**	**0 to –∞**
April to September	Hits, *H*	0	6	10	29	51	90	137	191	212	215	216
	Misses, *N*–*H*	0	0	0	2	4	13	42	166	391	589	682
	Insertions, *I*	0	0	1	4	5	8	9	9	9	9	9
	Total instances, *N*	0	6	10	31	55	103	179	357	603	804	898
	Accuracy	NA	100.0	90.0	80.6	83.6	79.6	71.5	51.0	33.7	25.6	23.1
	Accuracy, Avg.	NA	NA	NA	81.5	85.9	80.9	73.9	53.1	34.4	26.1	23.4
	Accuracy, SD	NA	NA	NA	17.6	15.0	19.2	18.6	18.6	10.4	7.7	6.8
	Correct	NA	100.0	100.0	93.5	92.7	87.4	76.5	53.5	35.2	26.7	24.1
	Correct, Avg.	NA	NA	NA	92.1	92.4	87.2	78.2	55.6	35.9	27.3	24.5
	Correct, SD	NA	NA	NA	13.7	8.9	11.7	14.3	16.9	9.3	6.9	6.1
	Missed	NA	0.0	0.0	6.5	7.3	12.6	23.5	46.5	64.8	73.3	75.9
	Missed, Avg.	NA	NA	NA	7.9	7.6	12.8	21.8	44.4	64.1	72.7	75.5
	Missed, SD	NA	NA	NA	13.7	8.9	11.7	14.3	16.9	9.3	6.9	6.1
	Precision	NA	100.0	90.9	87.9	91.1	91.8	93.8	95.5	95.9	96.0	96.0
	Precision, Avg.	NA	NA	NA	91.5	93.8	93.5	94.7	95.4	95.6	95.6	95.6
	Precision, SD	NA	NA	NA	11.4	8.5	10.2	8.6	7.8	7.5	7.5	7.5
