# Supplementary material for: Automated Sound Recognition Provides Insights into the Behavioral Ecology of a Tropical Bird
Source: PLoS One. 2017 Jan 13;12(1):e0169041. doi: 10.1371/journal.pone.0169041 (PMC5235375; doi:10.1371/journal.pone.0169041)
Supplement: S1 Fig — Landsat images by courtesy of the U.S. Geological Survey (http://landsatlook.usgs.gov): A = 4 May, B = 5 Jun., C = 7 Jul., D = 8 Aug., E = 24 Aug., and F = 25 Sep.; in 2013 no cloud-free images were available before and after these dates, including the period of maximum water levels in February/March 2013. (PDF) [file pone.0169041.s014.pdf]

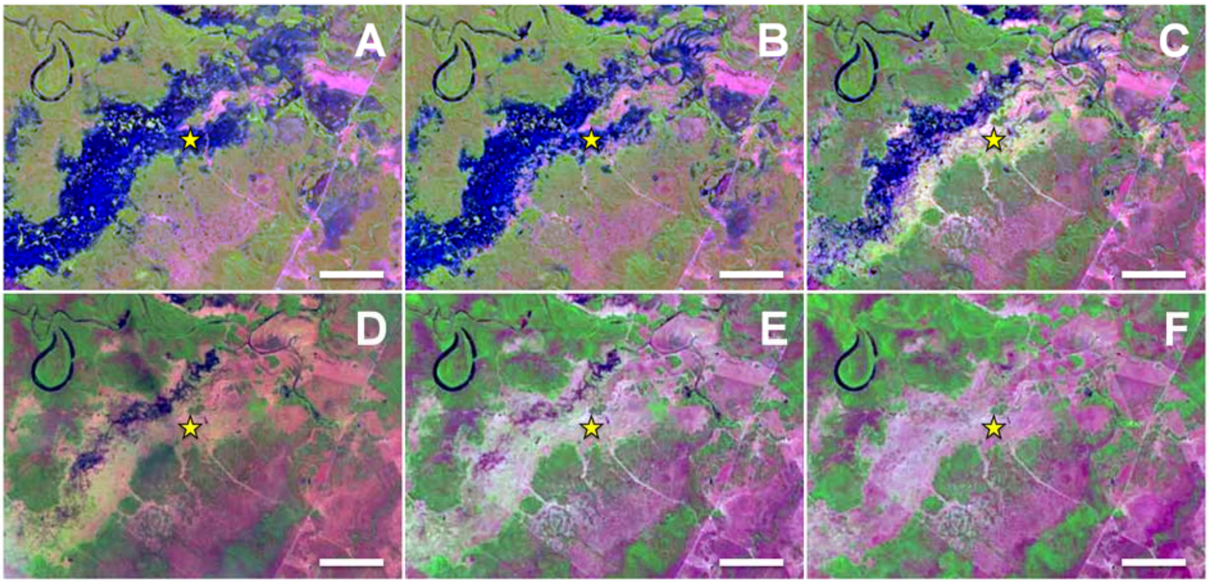

S1 Fig. Fazenda Pouso Alegre area in the northern Pantanal, municipality of Poconé, Mato Grosso, Brazil, during the *Vanellus chilensis* breeding season in 2013. Landsat images by courtesy of the U.S. Geological Survey (http://landsatlook.usgs.gov): A = 4 May, B = 5 Jun., C = 7 Jul., D = 8 Aug., E = 24 Aug., and F = 25 Sep.; in 2013 no cloud-free images were available before and after these dates, including the period of maximum water levels in February/March 2013. The continuous audio recordings analyzed in this study were collected at recording station PPA001 (yellow star) between 29 October 2012 and 26 October 2013. The white yardsticks correspond to a distance of 2 km. “Stretch 3 Std Dev” enhancement brightens the images without changing their colors: blue = expanses of water; whitish to pinkish areas = soil with sparse vegetation, mostly dried-out and overgrazed pastures; and green = verdant grasslands and woody vegetation. The green areas in image F represent mostly forests and dense shrubby vegetation. The Transpantaneira road (MT-060) is the linear structure forming a diagonal in the right third of the images.
