# Supplementary material for: Automated Sound Recognition Provides Insights into the Behavioral Ecology of a Tropical Bird
Source: PLoS One. 2017 Jan 13;12(1):e0169041. doi: 10.1371/journal.pone.0169041 (PMC5235375; doi:10.1371/journal.pone.0169041)
Supplement: S2 Fig — A: flooded cattle pasture with macrophytes at the height of the flood season. The water level at PPA001 was about 65 cm on that date (24 Feb. 2013). B: dry savannah-like cattle pasture at PPA001 at the end of the V. chilensis breeding season (2 Aug. 2013). (PDF) [file pone.0169041.s015.pdf]

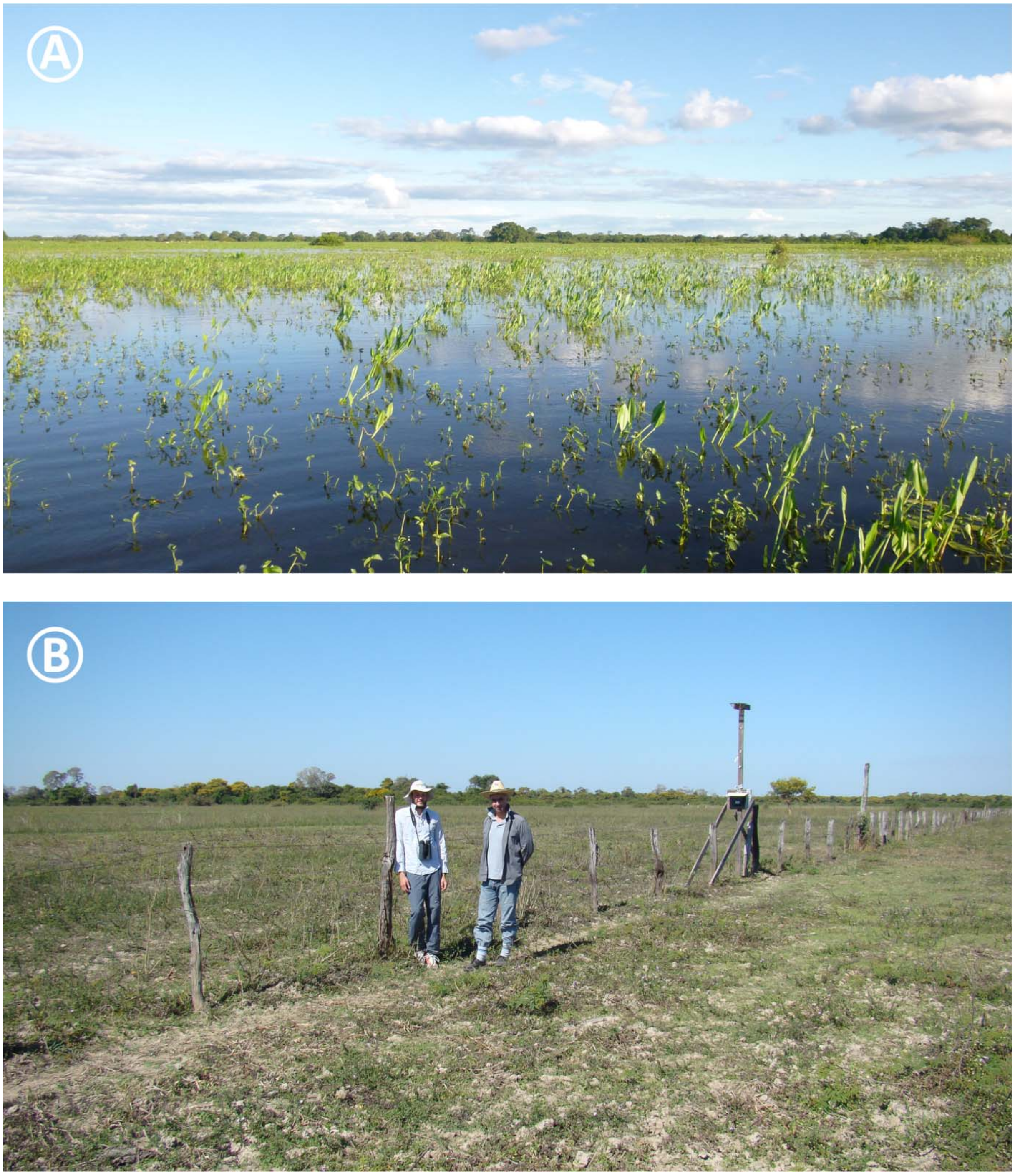

S2 Fig. *Vanellus chilensis* habitat near recording station PPA001 in the Fazenda Pouso Alegre area. A: flooded cattle pasture with macrophytes at the height of the flood season. The water level at PPA001 was about 65 cm on that date (24 Feb. 2013). B: dry savannah-like cattle pasture at PPA001 at the end of the *V. chilensis* breeding season (2 Aug. 2013). The SM2+ recorder with the omnidirectional SMX-II microphone to the left is mounted in the middle and the acrylic glass pane of the night flight call (NFC) microphone on the top of the 3.5-m high wooden construction (upper right quarter of the image). In the present study we processed only channel A, corresponding to the left microphone (i.e. SMX-II). Images by courtesy of CO.BRA/INAU (http://cobra.ic.ufmt.br). *Cf.* S1 Fig, yellow star marks recording site PPA001.
