# Supplementary material for: Automated Sound Recognition Provides Insights into the Behavioral Ecology of a Tropical Bird
Source: PLoS One. 2017 Jan 13;12(1):e0169041. doi: 10.1371/journal.pone.0169041 (PMC5235375; doi:10.1371/journal.pone.0169041)
Supplement: S3 Fig — (PDF) [file pone.0169041.s016.pdf]

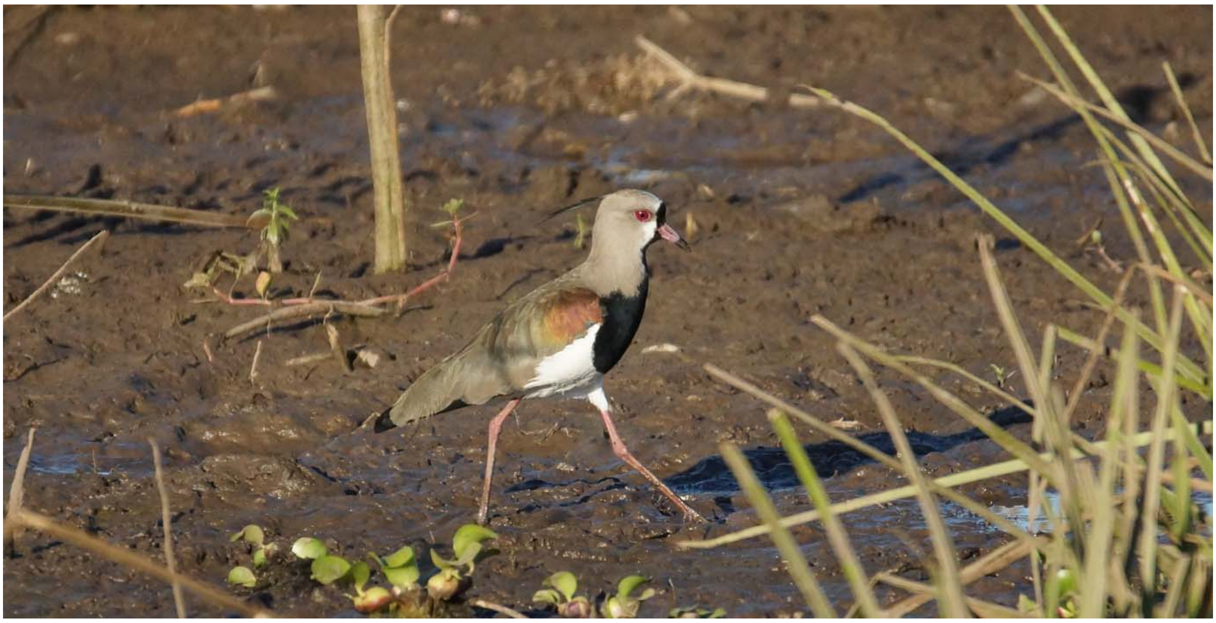

S3 Fig. *Vanellus chilensis lampronotus* searching for food at a muddy lake margin in the Fazenda Pouso Alegre area (18 Jul. 2012). Image by courtesy of CO.BRA/INAU (http://cobra.ic.ufmt.br).
