# Supplementary material for: Automated Sound Recognition Provides Insights into the Behavioral Ecology of a Tropical Bird
Source: PLoS One. 2017 Jan 13;12(1):e0169041. doi: 10.1371/journal.pone.0169041 (PMC5235375; doi:10.1371/journal.pone.0169041)
Supplement: S4 Fig — Cf. Fig 3, Table 3, and S2 Table. (PDF) [file pone.0169041.s017.pdf]

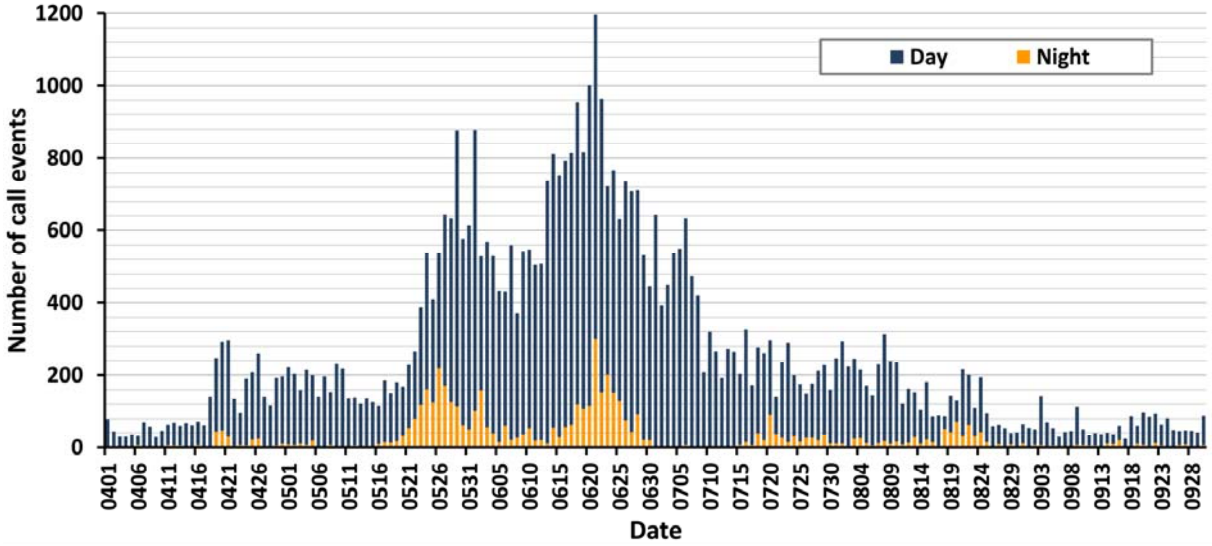

S4 Fig. Uncorrected *Vanellus chilensis* cumulative daily acoustic activity for the period April to September 2013 (*cf.* Fig 3, Table 3, and S1 Table).
