# Supplementary material for: Automated Sound Recognition Provides Insights into the Behavioral Ecology of a Tropical Bird
Source: PLoS One. 2017 Jan 13;12(1):e0169041. doi: 10.1371/journal.pone.0169041 (PMC5235375; doi:10.1371/journal.pone.0169041)
Supplement: S1 Table — See Table 3 for acoustic activity periods. (PDF) [file pone.0169041.s018.pdf]

S1 Table. Cumulated monthly daytime and nighttime detections of *Vanellus chilensis* call events and calculation of precision for the period April to September 2013.

	Month						
**Detection category**	**April**	**May**	**June**	**July**	**August**	**September**	**Total**
Raw detections daytime	3159	7663	18 151	8899	4078	1642	43 592
Raw detections nighttime	256	1444	2347	468	619	153	5287
Total raw detections	3415	9107	20 498	9367	4697	1795	48 879
N random sample, recordings	124	176	335	171	131	126	1063
N random sample, detections	150	200	400	200	150	150	1250
Proportion total detections (%)	4.4	2.2	2.0	2.1	3.2	8.4	2.6
N correct random detections	72	143	365	193	138	103	1014
N false random detections	78	54	32	6	11	47	228
N excluded random detections	0	3	3	1	1	0	8
N valid random detections	150	197	397	199	149	150	1242
Precision (%)	48.0	72.6	91.9	97.0	92.6	68.7	81.6

See Table 1 for cumulated monthly raw detections for the period November 2012 to October 2013 and Table 3 for acoustic activity periods of the breeding cycle (Apr.-Sep. 2013).
