# Supplementary material for: Automated Sound Recognition Provides Insights into the Behavioral Ecology of a Tropical Bird
Source: PLoS One. 2017 Jan 13;12(1):e0169041. doi: 10.1371/journal.pone.0169041 (PMC5235375; doi:10.1371/journal.pone.0169041)
Supplement: S2 Table — The false positive rates (= 100 –Precision [%]) were used to correct the number of diurnal and nocturnal detections in Fig 3. (PDF) [file pone.0169041.s019.pdf]

S2 Table. Identified sound sources in a random sample of 1250 detections, drawn from 48 879 *Vanellus chilensis* recognizer detections in soundscapes recorded at monitoring station PPA001, Pantanal region, Brazil, between April and September 2013.

Species / sound source	Family	Detection category[1]	Acoustic activity periods (no., mmdd)								Sum
			1	2	3	4	5	6	7	8	
			0401–0425	0426–0520	0521–0531	0601–0612	0613–0630	0701–0731	0801–0831	0901–0930	
a) Correct detections of target species											
Vanellus chilensis	Charadriidae	correct	39	88	88	93	272	193	138	103	1014
b) Ambiguous detections of target and competing species (alphabetic order)											
Vanellus chilensis, juv.?	Charadriidae	excluded						1			1
Vanellus chilensis / *Amazona aestiva*	Charadriidae / Psittacidae	excluded							1		1
Vanellus chilensis / *Aramus guarauna*	Charadriidae / Aramidae	excluded				1	1				2
Vanellus chilensis / *Dendrocygna autumnalis*	Charadriidae / Anatidae	excluded			1						1
Vanellus chilensis / frog	Charadriidae / unknown	excluded			1						1
Vanellus chilensis / *Himantopus melanurus*	Charadriidae / Recurvirostridae	excluded			1						1
Vanellus chilensis / *Jacana jacana*	Charadriidae / Jacanidae	excluded					1				1
c) False detections of identified competing animal sounds (alphabetic order)											
Amazona aestiva	Psittacidae	false					1		1	1	3
Anthus lutescens	Motacillidae	false							2		2
Aramus guarauna	Aramidae	false	35	9	6	6	8	1		1	66
Aramus guarauna / cattle	Aramidae / Bovidae	false	1								1
Aramus guarauna / *Myiopsitta monachus*	Aramidae / Psittacidae	false	1								1
Dendrocygna autumnalis	Anatidae	false		9	12	6					27
Furnarius rufus	Furnariidae	false					1	1			2
Furnarius rufus / *Myiopsitta monachus*	Furnariidae / Psittacidae	false					1				1
Guira guira	Cuculidae	false	1				1	3	1	6	12
Himantopus melanurus	Recurvirostridae	false		9	1						10
Jacana jacana	Jacanidae	false	24	8							32
Jacana jacana / *Dendrocygna autumnalis*	Jacanidae / Anatidae	false			1						1
Rostrhamus sociabilis / *Myiopsitta monachus*	Accipitridae / Psittacidae	false	1								1
Phaetusa simplex	Laridae	false					1				1
Myiopsitta monachus	Psittacidae	false	3			1	3	1	3	11	22
Primolius auricollis	Psittacidae	false								1	1
Theristicus caudatus	Threskiornithidae	false							1		1
Pitangus sulphuratus	Tyrannidae	false	2	1			3		1	19	26
Pitangus sulphuratus / *Jacana jacana*	Tyrannidae / Jacanidae	false	1								1
d) False detections of unidentified competing animal sounds (alphabetic order)											
Bat, unidentified species	unknown	false							1	2	3
Bird, unidentified species	unknown	false								1	1
Frog, unidentified species	unknown	false			4						4
Frog / *Dendrocygna autumnalis*	unknown / Anatidae	false			2						2
e) False detections of competing abiotic sounds (alphabetic order)											
Data saving noise[2]		false	1								1
Gust of wind		false							1	5	6

S2 Table. Continued.

			Acoustic activity periods (no., mmdd)								
			1	2	3	4	5	6	7	8	
**Species / sound source**	**Family**	**Detection category[1]**	**0401–0425**	**0426–0520**	**0521–0531**	**0601–0612**	**0613–0630**	**0701–0731**	**0801–0831**	**0901–0930**	**Sum**
N correct detections			39	88	88	93	272	193	138	103	1014
N false detections			70	36	26	13	19	6	11	47	228
N excluded detections					3	1	2	1	1		8
Grand total			109	124	117	107	293	200	150	150	1250
Total valid detections[3]			109	124	114	106	291	199	149	150	1242
Precision (%)[4]			35.8	71.0	77.2	87.7	93.5	97.0	92.6	68.7	81.6

The false positive rates (= 100 – Precision [%]) were used to correct the number of diurnal and nocturnal detections (*cf.* Fig 3). See S1 Table for monthly precision rates.

[1] All randomly selected detections were assessed and identified by a bird sound expert and counted as correct (true positive) when the start/end timestamps exclusively embraced *Vanellus chilensis* sounds and as false (false positive) when the detection only contained non-target signals. Some detections contained sounds of *V. chilensis* and competing species. These detections were (a) labelled as true positive when most of the audio signals were emitted by the target species; (b) rated as false positive when non-target species were the dominant sound source; and (c) excluded from analysis when it was unclear whether the detection was triggered by sounds of *V. chilensis* or another species.

[2] Electronic noise generated by the SM2+ recorder while saving the audio data to the SSD card.

[3] Total valid detections = grand total – N excluded.

[4] See Materials and Methods section for formula.
