# Supplementary material for: Automated Sound Recognition Provides Insights into the Behavioral Ecology of a Tropical Bird
Source: PLoS One. 2017 Jan 13;12(1):e0169041. doi: 10.1371/journal.pone.0169041 (PMC5235375; doi:10.1371/journal.pone.0169041)
Supplement: S3 Table — See S1 Appendix for performance assessment results. Double hits refer to repeated detections of the same lapwing call series and were omitted in subsequent analyses. The weakest event in dB refers to the loudest note of the weakest V. chilensis call event detected by the automated recognizer among correct hits and double hits. (PDF) [file pone.0169041.s020.pdf]

S3 Table. Validation library: soundscapes annotated for the performance assessment of the *Vanellus chilensis* recognizer.

Month	Soundscape	Daytime	N target[1]	Detections	False positive	Correct hits	Double hits	Double hit rate (%)	Weakest event (dB)
April	BRMTAPPPA001A20130401T065409.wav	Day	20	12	0	9	3	25.0	–43.0
	BRMTAPPPA001A20130412T064931.wav	Day	29	5	0	4	1	20.0	–39.0
	BRMTAPPPA001A20130418T173813.wav	Day	55	16	0	14	2	12.5	–41.0
	BRMTAPPPA001A20130426T063906.wav	Day	31	12	0	8	4	33.3	–37.0
	Sum		135	45	0	35	10	NA	NA
	Average		33.8	11.3	0.0	8.8	2.5	22.7	–40.0
	SD		15.0	4.6	0.0	4.1	1.3	8.7	2.6
May	BRMTAPPPA001A20130504T171206.wav	Day	38	10	3	6	1	14.3	–41.0
	BRMTAPPPA001A20130512T061858.wav	Day	28	11	1	6	4	40.0	–31.0
	BRMTAPPPA001A20130516T093414.wav	Day	18	10	0	5	5	50.0	–35.0
	BRMTAPPPA001A20130529T144441.wav	Day	54	11	2	8	1	11.1	–34.0
	Sum		138	42	6	25	11	NA	NA
	Average		34.5	10.5	1.5	6.3	2.8	28.8	–35.3
	SD		15.4	0.6	1.3	1.3	2.1	19.1	4.2
June	BRMTAPPPA001A20130606T091946.wav	Day	43	12	1	8	3	27.3	–35.0
	BRMTAPPPA001A20130611T141505.wav	Day	40	11	0	11	0	0.0	–41.0
	BRMTAPPPA001A20130616T103315.wav	Day	49	11	0	9	2	18.2	–41.0
	BRMTAPPPA001A20130625T034348.wav	Night	16	11	0	6	5	45.5	–32.0
	Sum		148	45	1	34	10	NA	NA
	Average		37.0	11.3	0.3	8.5	2.5	22.7	–37.3
	SD		14.5	0.5	0.5	2.1	2.1	18.9	4.5
July	BRMTAPPPA001A20130703T170509.wav	Day	60	13	0	11	2	15.4	–42.0
	BRMTAPPPA001A20130710T060216.wav	Day	51	10	0	8	2	20.0	–34.5
	BRMTAPPPA001A20130716T175252.wav	Day	52	12	0	6	6	50.0	–51.0
	BRMTAPPPA001A20130728T070420.wav	Day	34	11	0	10	1	9.1	–41.0

S3 Table. Continued.

Month	Soundscape	Daytime	N target[1]	Detections	False positive	Correct hits	Double hits	Double hit rate (%)	Weakest event (dB)
	Sum		197	46	0	35	11	NA	NA
	Average		49.3	11.5	0.0	8.8	2.8	23.6	–42.1
	SD		10.9	1.3	0.0	2.2	2.2	18.1	6.8
August	BRMTAPPPA001A20130801T073420.wav	Day	41	14	0	10	4	28.6	–39.0
	BRMTAPPPA001A20130812T054141.wav	Day	54	12	0	10	2	16.7	–39.0
	BRMTAPPPA001A20130818T090439.wav	Day	18	10	0	9	1	10.0	–44.0
	BRMTAPPPA001A20130824T044939.wav	Night	22	10	0	10	0	0.0	–45.0
	Sum		135	46	0	39	7	NA	NA
	Average		33.8	11.5	0.0	9.8	1.8	13.8	–41.8
	SD		16.8	1.9	0.0	0.5	1.7	12.0	3.2
September	BRMTAPPPA001A20130901T054911.wav	Day	43	11	1	7	3	30.0	–42.5
	BRMTAPPPA001A20130906T180743.wav	Day	15	12	0	5	7	58.3	–51.0
	BRMTAPPPA001A20130911T064044.wav	Day	27	10	0	10	0	0.0	–39.0
	BRMTAPPPA001A20130916T060354.wav	Day	24	11	1	6	4	40.0	–35.0
	BRMTAPPPA001A20130926T170626.wav	Day	14	11	0	11	0	0.0	–28.0
	BRMTAPPPA001A20130930T121338.wav	Day	22	11	0	9	2	18.2	–41.0
	Sum		145	66	2	48	16	NA	NA
	Average		24.2	11.0	0.3	8.0	2.7	24.4	–39.4
	SD		10.5	0.6	0.5	2.4	2.7	23.0	7.7
Apr. to Sep.	Sum		898	290	9	216	65	NA	NA
	Average		34.5	11.2	0.3	8.3	2.5	22.8	–39.3
	SD		14.6	1.8	0.7	2.4	1.9	16.7	5.4

[1]N target = number of expert-confirmed *V. chilensis* call events.

See S1 Appendix for performance assessment results. Double hits refer to repeated detections of the same lapwing call series and were omitted in subsequent analyses. The weakest event in dB refers to the loudest note of the weakest *V. chilensis* call event detected by the automated recognizer among correct hits and double hits.
