# Supplementary material for: Automated Sound Recognition Provides Insights into the Behavioral Ecology of a Tropical Bird
Source: PLoS One. 2017 Jan 13;12(1):e0169041. doi: 10.1371/journal.pone.0169041 (PMC5235375; doi:10.1371/journal.pone.0169041)
Supplement: S4 Table — Table 2 presents the cumulated numbers (Apr. to Sep. 2013). (PDF) [file pone.0169041.s021.pdf]

S4 Table. Validation Library: monthly number of confirmed and unconfirmed sound events according to the loudest call amplitude in *Vanellus chilensis* vocalizations.

Month / Range (dB)	N target[1]	N unconfirmed	N total	% unconfirmed
April				
0 to –5	0	0	0	NA
0 to –10	0	0	0	NA
0 to –15	0	0	0	NA
0 to –20	3	0	3	0
0 to –25	6	0	6	0
0 to –30	12	0	12	0
0 to –35	19	0	19	0
0 to –40	44	0	44	0
0 to –45	84	1	85	1.2
0 to –50	122	11	133	8.3
0 to –∞	135	59	194	30.4
May				
0 to –5	0	0	0	NA
0 to –10	2	0	2	0
0 to –15	3	0	3	0
0 to –20	5	0	5	0
0 to –25	13	0	13	0
0 to –30	19	0	19	0
0 to –35	34	1	35	2.9
0 to –40	61	6	67	9.0
0 to –45	102	22	124	17.7
0 to –50	125	48	173	27.7
0 to –∞	138	112	250	44.8
June				
0 to –5	0	0	0	NA
0 to –10	2	0	2	0
0 to –15	2	0	2	0
0 to –20	7	0	7	0
0 to –25	10	0	10	0
0 to –30	21	0	21	0
0 to –35	38	0	38	0
0 to –40	65	1	66	1.5
0 to –45	107	6	113	5.3
0 to –50	133	13	146	8.9
0 to –∞	148	52	200	26.0

S4 Table. Continued.

Month / Range (dB)	N target[1]	N unconfirmed	N total	% unconfirmed
July				
0 to –5	0	0	0	NA
0 to –10	0	0	0	NA
0 to –15	1	0	1	0
0 to –20	2	0	2	0
0 to –25	5	0	5	0
0 to –30	12	0	12	0
0 to –35	31	0	31	0
0 to –40	77	2	79	2.5
0 to –45	121	3	124	2.4
0 to –50	177	15	192	7.8
0 to –∞	197	81	278	29.1
August				
0 to –5	0	0	0	NA
0 to –10	0	0	0	NA
0 to –15	0	0	0	NA
0 to –20	3	0	3	0
0 to –25	5	0	5	0
0 to –30	11	0	11	0
0 to –35	21	0	21	0
0 to –40	49	1	50	2.0
0 to –45	97	3	100	3.0
0 to –50	121	13	134	9.7
0 to –∞	135	60	195	30.8
September				
0 to –5	0	0	0	NA
0 to –10	2	0	2	0
0 to –15	4	0	4	0
0 to –20	11	0	11	0
0 to –25	16	0	16	0
0 to –30	28	0	28	0
0 to –35	36	0	36	0
0 to –40	61	0	61	0
0 to –45	92	3	95	3.2
0 to –50	126	20	146	13.7
0 to –∞	145	90	235	38.3

[1]N target = number of expert-confirmed *V. chilensis* call events.

Table 2 presents the cumulated numbers for April to September 2013.
